# Deciphering Steroidal and Aporphine Alkaloids as Antileukemic Agents by Approaches of Molecular Networking and Metabolomics

**DOI:** 10.1021/acsomega.4c10160

**Published:** 2025-03-06

**Authors:** Suni Liu, Katyuce Souza Farias, Vanessa Samudio Santos Zanuncio, Geraldo Alves Damasceno Júnior, Flávio Macedo Alves, Edgar J. Paredes-Gamero, Kamylla Fernanda Souza de Souza, Lucas Roberto Pessatto, Heron Fernandes Vieira Torquato, Carlos Alexandre Carollo, Denise Brentan Silva

**Affiliations:** 1 Faculty of Pharmaceutical Sciences, Food and Nutrition (FACFAN), Laboratory of Natural Products and Mass Spectrometry (LaPNEM), Federal University of Mato Grosso do Sul, Campo Grande, Mato Grosso do Sul 79070-900, Brazil; 2 Laboratory of Botany, Institute of Biosciences (INBIO), Federal University of Mato Grosso do Sul, Campo Grande, Mato Grosso do Sul 79070-900, Brazil; 3 Laboratory of Molecular Biology and Cell Cultures, Faculty of Pharmaceutical Sciences, Food and Nutrition (FACFAN), Federal University of Mato Grosso do Sul, 79070-900, Mato Grosso do Sul Campo Grande, Brazil; 4 Biochemistry Department, Federal University of São Paulo, São Paulo, SP 04044-020, Brazil

## Abstract

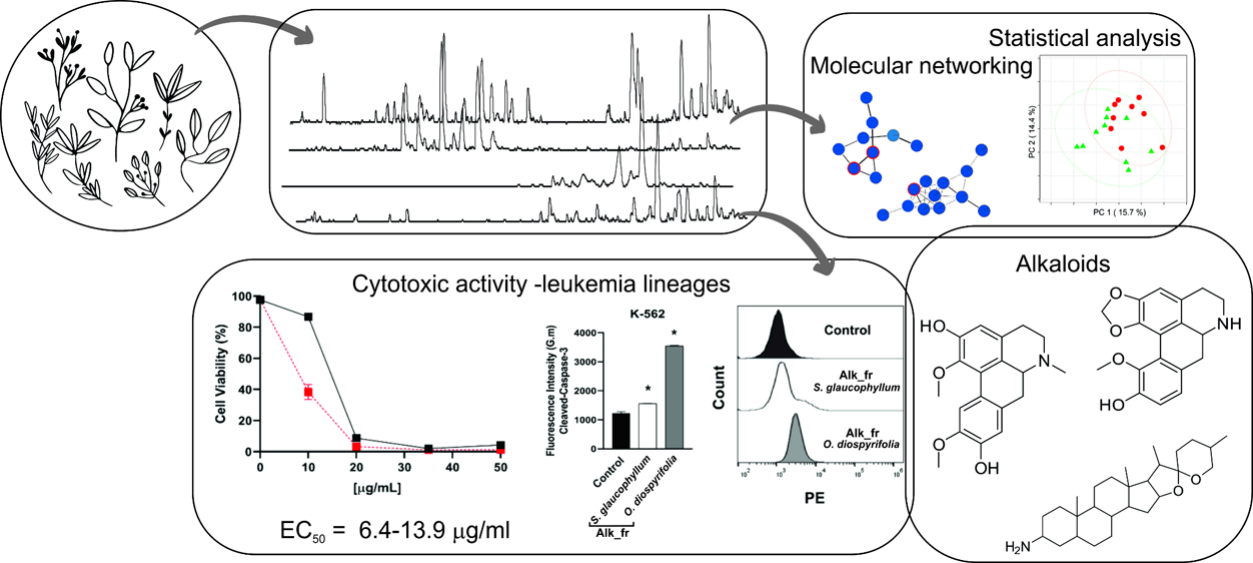

The chemodiversity of plants is a valuable resource for drug discovery, and its combination with modern approaches can reduce the time consumption for bioactive metabolite discovery. This study aimed to evaluate the chemical constituents from 18 plant species of different families against leukemia cancer cells and the application of statistical analysis from metabolomic data and molecular networking for the prediction of bioactive metabolites. The samples, extracted by an accelerated solvent extractor using ethanol and water 7:3 (v/v), were analyzed by LC-DAD-MS and evaluated against leukemia cancer cells (Kasumi-1, KG-1, and K-562). Chemical data were aligned, analyzed by statistics, and applied to create the molecular network. *Sesbania virgata*, *Aeschynomene denticulata*, *Erythroxylum angiufugum*, *Psidium guineense*, *Astronium fraxinifolium*, *Coccoloba ochreolata*, *Solanum glaucophyllum* (*S. glaucophyllum*), and *Paullinia pinnata* inhibited K-562 leukemia cancer cell viability by approximately 70% at 100 μg/mL, while *Ocotea diospyrifolia* showed 35% inhibition for the KG-1 lineage. Alkaloid fractions from *S. glaucophyllum* and *O. diospyrifolia* revealed EC_50_ values ranging from 13.9 to 6.4 μg/mL for K-562 and KG-1 cell lines, effectively inducing cell death with apoptotic characteristics, membrane integrity loss, and signs of late apoptosis. The molecular networking was essential and crucial to complement the statistical analysis, which was performed from 430 features and targeted steroidal and aporphine alkaloids. Boldine revealed EC_50_ values of 46, 116, and 145 μM for Kasumi, KG-1, and K-562 cancer cell lines, respectively. The findings marked the relevance of a broader chemical data analysis to predict bioactive compounds, emphasizing potential benefits in the search for metabolites against leukemia cancer cells, particularly steroidal and aporphine alkaloids.

## 1 Introduction

Leukemia is a hematologic cancer that affects children, adults, adolescents, and elderly.^1^ In 2019, the global incidence of new leukemia cases was 643,579, while 474,924 deaths were registered. Additionally, 720,168 new leukemia cases are estimated in 2030.^2^ Leukemia can be described in four main subtypes according to the speed (acute or chronic) and blood cell type (myeloid or lymphoid).^2,3^

Leukemia induces an abnormal increase in leukocytes in the bone marrow or peripheral blood, resulting in cell populations at various differentiation stages, including leukemic stem cells, which exhibit low proliferation rates and a quiescent stage. These cells represent a problem for conventional treatments since the drugs act mainly against cells with high proliferation rates. Furthermore, leukemic stem cells are closely associated with disease recurrence, making them an important target for the development of new drugs, which are highly required.^4,5^

Natural products are a valuable source for the discovery of new bioactive compounds, representing approximately 50% of the approved drug candidates between 1981 and 2019. Notably, among the small compound anticancer drugs approved in this period, 76.7% are derived from natural products that highlight their importance and critical role in the development of new therapeutic agents, for example, the anticancer drugs taxol, vinblastine, and vincristine.^6^

Metabolomics and molecular networking have been successfully applied to suggest bioactive metabolites from natural product samples. These approaches accelerate the search and development of new drug candidates, promoting a rapid annotation of metabolic classes and target prediction.^7^ These strategies provide more holistic chemical information about the composition of extracts and fractions and the potential bioactive compounds.^8^ Metabolomics and statistical analyses were applied, for example, to determine the bioactive compounds from Brazilian plant species (*Aspidosperma subincanum*, *Copaifera langsdorffii*, *Coussarea hydrangeifolia*, *Guarea guidonea*, and *Tapirira guianensis*) against resistant human melanoma cells. Flavonoids, galloylquinic acid derivatives, and diterpenes were correlated to cytotoxic activity.^9^ Although the prediction of bioactive compounds has been done, several phenolic metabolites were identified, which are widely known in the literature and cannot represent a good target for the development of anticancer drugs. In this context, molecular networking can be a valuable tool, organizing the MS and MS/MS data and grouping similar metabolites that further the selection of the bioactive metabolic classes.^10^

This study aimed to use metabolomics and molecular networking approaches to determine the chemical composition from extracts of Brazilian plant species and to suggest the bioactive metabolites against leukemia cancer cell lineages (Kasumi-1, KG-11, and K-562).

## 2 Materials and Methods

### 2.1 Sample Collection

The plant materials were collected in Mato Grosso do Sul state, Brazil, in biomes Cerrado and Pantanal. The plant species were taxonomically identified by Alves and Damasceno Júnior. All specimens were deposited in the herbarium (CGMS) of Federal University of Mato Grosso do Sul (UFMS), and information about the plants is summarized in Table S1 (Supporting Information). This study was registered in the National Genetic Heritage Management System (SisGen) under the number A63DB37. The parts used for extraction were fruits (FR), aerial parts (AP), leaves (LV), and roots (RO). The collected species and their used parts are the following: *Aeschynomene denticulata* Rudd (AP, Fabaceae), *Aspilia latissima* Malme (LV and RO, Asteraceae), *Astronium fraxinifolium* Schott (AP, Anacardiaceae), *Bauhinia mollis* (AP, Fabaceae), *Byttneria filipes* Mart. ex K.Schum. (AP, Malvaceae), *Centratherum punctatum* Cass. (AP, Asteraceae), *Coccoloba ochreolata* Wedd. (AP, Fabaceae), *Diospyros tetrasperma* Sw. (AP, Ebenaceae), *Echinodorus paniculatus* Micheli (AP, Alismataceae), *Erythroxylum anguifugum* Mart. (AP, Erythroxylaceae), *Lantana canescens* Kunth (AP, Verbenaceae), *Melanthera latifolia* (Gardner) Cabrera (AP, Asteraceae), *Ocotea diospyrifolia* (Meisn.) Mez (AP, Lauraceae), *Paullinia pinnata* L. (AP, Sapindaceae), *Psidium guineense* Sw. (AP, Myrtaceae), *Sesbania virgata* (Cav.) Pers. (AP, Fabaceae), *Solanum glaucophyllum* Desf. (FR, Solanaceae), *Tocoyena formosa* (Cham. & Schltdl.) K. Schum. (AP, Rubiaceae), and *Vitex cymosa* Bertero ex Spreng. (AP, Lamiaceae). The plants were dried in a circulation oven (40 °C) and powdered in a knife mill.

### 2.2 Preparation of Extracts and Fractions

The dried and powdered plant materials (fruits, aerial parts, leaves, and roots) were extracted by an accelerated solvent extractor (ASE 150, Dionex) using ethanol and water 7:3 (v/v) at 100 °C, 1600 psi, a single cycle, a 5 min static time, a 60% rinse volume, and a 50 s purge time. These plant materials were previously cleaned using hexane and acetone 8:2 (v/v) to remove the wax constituents by ASE. All the extracts were concentrated by a rotatory evaporator, subsequently lyophilized, and kept at −20 °C until the biological and chemical evaluations. The hydroethanolic extracts from *O. diospyrifolia* and *S. glaucophyllum* were submitted to alkali-acid extractions to obtain the enriched alkaloid fractions (Alk_fr), as described by Sharma and collaborators,^11^ and they yielded the fractions with yields of 0.15 and 0.03%, respectively.

### 2.3 Chemical Analysis by LC-DAD-MS

The extracts were analyzed by liquid chromatography coupled to a diode array detector and a high-resolution mass spectrometer (LC-DAD-MS). A Shimadzu UFLC chromatograph (Tokyo, Japan) equipped with two LC20AD pumps, a SIL-20A autoinjector, an SPD-M20A diode array detector (DAD), a CBM-20A controller, and a CTO-20A oven was used. This equipment was also coupled to a MicrOTOF-Q III mass spectrometer (Bruker Daltonics) with electrospray ionization (ESI) and a QqTOF analyzer (quadrupole and time-of-flight).

For chromatographic analysis, a Kinetex C18 column (2.6 μm, 100 Å, 150 × 2.1 mm, Phenomenex) was applied, which was kept at 50 °C during the analysis, and the flow rate was 0.3 mL/min. The mobile phase was composed of acetonitrile (B) and ultrapure water (A), both containing formic acid 0.1% (v/v). The gradient elution profile was programmed to 0–2 min 3% B, 2–25 min 3–25% B, 25–40 min 25–80% B, and 40–43 min 80% B. The samples were analyzed in positive and negative ion modes, and the MS parameters were the capillary voltages of 2,500 and 3,500 kV for positive and negative ion modes, respectively. Nitrogen was applied as a nebulizer (4 bar), drying (9 L/min), and collision gas.

The 19 polar extracts were solubilized in methanol and water 6:4 (v/v) at a concentration of 2 mg/mL and filtered on 0.22 μm PTFE syringe filters (Millex, Millipore), and 1 μL of each sample was injected into the chromatographic system.

### 2.4 Statistical Processing and Analysis of Metabolomic Data

The raw data were converted to .cdf and, subsequently, were aligned and reduced by software MetAlign and MSClust, respectively. Univariate and multivariate statistical analyses were performed by Metaboanalyst 6.0. The biological activity data were included in the data sets to correlate the chemical and biological data and to suggest the bioactive compounds. The annotation was based on UV, MS, and MS/MS spectral data compared to literature data, as well as a spectral comparison of mass spectrometry data deposited in GNPS (https://gnps.ucsd.edu). The molecular formulas of compounds were determined considering errors and mSigma values up to 8 ppm and 30, respectively.

The data were log-transformed (base 10) and used to obtain principal component analysis (PCA), and the hierarchical clustering (dendrogram) was constructed applying the Euclidean distance measure and clustering algorithm ward. A volcano plot based on the fold change and a *p*-value threshold of 0.05 were also obtained.

### 2.5 Molecular Networking and Annotation

LC-MS and MS/MS data were converted to mzXML by MsConvert. Subsequently, these data were applied to produce the molecular networking by the GNPS platform, and the molecular networking was processed and edited in Cytoscape 3.9.1.

The molecular networking was constructed considering five fragment ions from MS/MS spectra, and the mass tolerances of precursor and fragment ions were considered 0.03 and 0.08 Da, respectively. The molecular network was created considering cosine greater than 0.6 and five ions combined. It is available in the GNPS platform at the link https://gnps.ucsd.edu/ProteoSAFe/status.jsp?task=ab38a049b1314471bf42eea12f88fd31. Additionally, the raw data were processed using MZmine software (version 4.5.0), following a series of steps: mass detection, chromatogram building, chromatogram deconvolution, deisotoping, alignment using the join aligner, blank subtraction from the feature list, feature finding, linear normalization, and spectral library searching. Detailed parameters for each step are provided in the Supporting Information. The MS1 data were exported in .csv format, while the MS2 data were prepared for upload to the GNPS platform and to obtain the feature-based molecular networking (FBMN) (https://gnps.ucsd.edu/ProteoSAFe/status.jsp?task=9dc8656aa292409e8337da1d35c4eb82). Compound annotation was performed using spectral library matching within MZmine, complemented by manual annotation based on spectral data comparisons of the literature-reported data.

### 2.6 Cell Cultures

Human leukemic cell lines (K-562, Kasumi-1, and KG-1) were obtained from the American Type Culture Collection (ATCC). K-562 was maintained in a Roswell Park Memorial Institute (RPMI 1640) medium (Sigma-Aldrich, Germany) supplemented with 10% FBS. The cells were cultured in Iscove’s modified Dulbecco’s medium (IMDM) supplemented with 20% fetal bovine serum (FBS). The cell lines were grown in media containing 100 U/mL penicillin (Sigma-Aldrich, Germany) and 100 μg/mL streptomycin (Sigma-Aldrich, Germany) in a humidified incubator at 37 °C with 5% CO_2_. The passage numbers for all leukemic cell lines ranged between 3 and 6.

### 2.7 Cytotoxicity Effects on Leukemia Cell Lines

The cytotoxicity assay was performed by the AlamarBlue assay method (AlamarBlue, Biosource, Camarillo, CA, USA).^12,13^ The extracts, fractions, and boldine (Sigma-Aldrich) were evaluated against K-562 (chronic myeloid leukemia), KG-1 (acute myeloid leukemia), and Kasumi-1 (acute myeloblastic leukemia) cell lines. The plant extracts and fractions, previously lyophilized and homogenized, were initially evaluated using 10 and 100 μg/mL, and the alkaloid fractions from *O. diospyrifolia* and *S. glaucophyllum* were evaluated against K-562 and KG-1 leukemic cell lines treated with different concentrations (50, 35, 20, and 10 μg/mL).

The samples were resuspended in dimethyl sulfoxide (DMSO), diluted in culture medium (final concentration DMSO 0.4%), and added to the test plates. Cells (1 × 10^5^ cells/mL^–1^) were grown in 96-well microplates containing a supplemented medium and different concentrations of the extracts. After incubation for 24 h, 20 μL of resazurin solution (0.15 mg/mL) was added, and the plates were shaken briefly by a plate shaker. After the incubation for 4 h at 37 °C, the assay plates were shaken before measurements. The samples were measured at wavelengths of 570 and 600 nm by a microplate reader (Thermo Scientific Varioskan LUX). Each experiment was performed in triplicate. The percentage of growth for each cell line was calculated in the program for graphs and data analysis Prism, GraphPad 8.0.

### 2.8 Annexin V/7-AAD Flow Cytometry Assay

An Annexin V-FITC/7-AAD double staining assay was conducted to assess the proapoptotic effect of the alkaloid fractions from *O. diospyrifolia* and *S. glaucophyllum*. K-562 and KG-1 cells were seeded at a density of 10^5^ cells/mL in 96-well plates and treated with alkaloid fractions of *S. glaucophyllum* (13 μg/mL) and *O. diospyrifolia* (6 and 13 μg/mL to K-562 and KG-1, respectively). After 24 h, the cells were resuspended in an Annexin V binding buffer (0.14 M NaCl, 2.5 mM CaCl_2_, and 0.01 M HEPES, pH 7.4) and incubated at room temperature with 1 μL of Annexin V-FITC (Becton Dickinson, USA) and 5 μM DRAQ5 (Cell Signaling, USA) for 30 min. The analysis was carried out using a CytoFLEX flow cytometer (Beckman Coulter, USA) and FlowJo v10 software (Becton Dickinson, USA).

### 2.9 Intracellular Protein Labeling

Cells (10^5^/mL) were treated for 24 h with EC_50_ concentrations of the alkaloid fractions from *O. diospyrifolia* and *S. glaucophyllum*. After treatment, the cells were fixed with BD Cytofix (BD Biosciences, USA) for 15 min, washed with a Becton Dickinson Perm/wash buffer, and permeabilized with Perm Buffer III (Becton Dickinson, USA) for 30 min at room temperature. For intracellular protein labeling, the cells were incubated for 1 h with primary antibodies (cleaved caspase-3 PE catalog no. 550914, BD Biosciences, USA) and (phospho-histone H2A.X no. 9718, Cell Signaling, USA). Subsequently, an antirabbit IgG secondary antibody conjugated with Alexa Fluor 488 (Thermo Fisher Scientific, USA) was incubated for 40 min. Fluorescence was then measured using a CytoFLEX flow cytometer (Beckman Coulter, USA), and the data were analyzed using FlowJo v10 software (Becton Dickinson, USA). A total of 40,000 events were collected per sample, and protein analyses were conducted by quantifying the geometric mean (G.m).

### 2.10 Statistical Analysis

The results obtained in the *in vitro* assay of the cytotoxicity effect against the cell lines were expressed as means ± standard error of the mean (SEM) and compared with the controls by analysis of variance (ANOVA) followed by Tukey’s test (GraphPad Prism 5). The statistically significant *p*-value <0.05 was considered as a statistical difference.

## 3 Results and Discussion

### 3.1 Cytotoxicity Effects on Leukemia Cell Lines of Extracts

**Figure 1:**
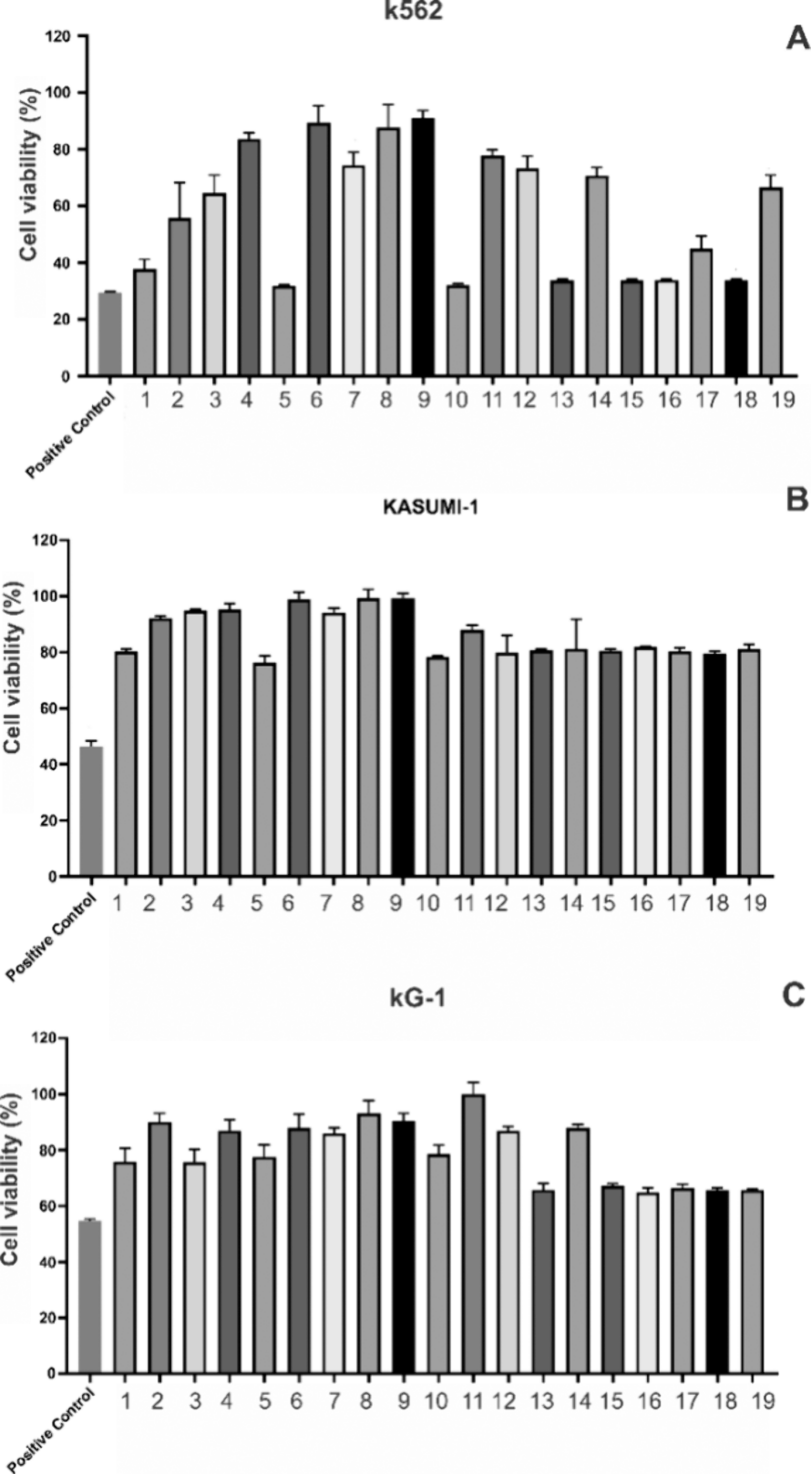
Cell proliferation and cytotoxic screening of the polar extracts on K-562 (A), Kasumi-1 (B), and KG-1 (C) leukemia cell lines at 100 μg/mL for 24 h. Extracts from the following species were evaluated: *Sesbania virgata* (1), *Centratherum punctatum* (2), *Lantana canescens* (3), *Melanthera latifolia* (4), *Aeschynomene denticulata* (5), *Echinodorus paniculatus* (6), *Byttneria filipes* (7), *Aspilia latissima* aerial parts (AP) (8), *A. latissima* roots (9), *Erythroxylum anguifugum* (10), *Tocoyena formosa* (11), *Diospyros tetrasperma* (12), *Psidium guineense* (13), *Vitex cymosa* (14), *Astronium fraxinifolium* (15), *Coccoloba ochreolata* (16), *Solanum glaucophyllum* (17), *Paullinia pinnata* (18), and *Ocotea diospyrifolia* (19). Doxorubicin was used as a positive control. Results were expressed as the mean ± standard deviation (SD).

The cytotoxicity of the extracts was initially evaluated against Kasumi-1, KG-1, and K-562 leukemia cell lines; the results are illustrated in Figure 1. The extracts were more active against the K-562 leukemia cell lineage, and the polar extracts that presented approximately a reduction of 60–70% of cell viability at 100 μg/mL were the following: *S. virgata*, *Aeschynomene denticulata* (*A.* denticulata), *E. anguifugum*, *P. guineense*, *A. fraxinifolium*, *C. ochreolata*, *S. glaucophyllum*, and *P. pinnata* (Figure 1A).

The extracts from *P. guineense*, *A. fraxinifolium*, *C. ochreolata*, *S. glaucophyllum*, *P. pinnata*, and *O. diospyrifolia* showed approximately 40% inhibition on the KG-1 leukemia cell line (Figure 1B), while the extracts exhibited lower activity against the Kasumi-1 leukemia cell lineage than the observed for the other lineages. The most active extracts against Kasumi-1 leukemia cells that revealed inhibition of about 20% were *S. virgata*, *E. anguifugum*, *A. denticulata*, *D. tetrasperma*, *P. guineense*, *V. cymosa*, *A. fraxinifolium*, *C. ochreolata*, *S. glaucophyllum*, *P. pinnata*, and *O. diospyrifolia* (Figure 1C). All the extracts were also evaluated against leukemia cell lines at 10 μg/mL, and they were inactive at this concentration except the extracts of *S. glaucophyllum* and *O. diospyrifolia* on the Kasumi-1 cell lineage that revealed a cell inhibition of 40% (Figure S1, Supporting Information).

Species from the Fabaceae, Erythroxylaceae, Myrtaceae, Anacardiaceae, Solanaceae, Sapindaceae, and Lauraceae families demonstrated activity against leukemia cell lines. These findings suggest that these plants can be promising sources of bioactive compounds for leukemia cells. However, modern approaches to generate comprehensive chemical profiles from samples are crucial to accelerate the identification of bioactive compounds, minimizing the selection of nonpromising targets or pan-assay interference compounds (PAINS).^9,10^

### 3.2 Chemical Analysis Metabolomics and Molecular Networking

The extracts were analyzed by LC-DAD-MS, and they were categorized into active and inactive extracts according to the cytotoxicity observed on the leukemic cancer cell lines (KG-1, Kasumi-1, and K-562). All of the chromatograms from the extracts are illustrated in Figure S2. The LC-MS data were processed, as described previously, and 480 entrances were listed. Statistical analyses were performed in the Metaboanalyst 6.0 platform.

**Figure 2:**
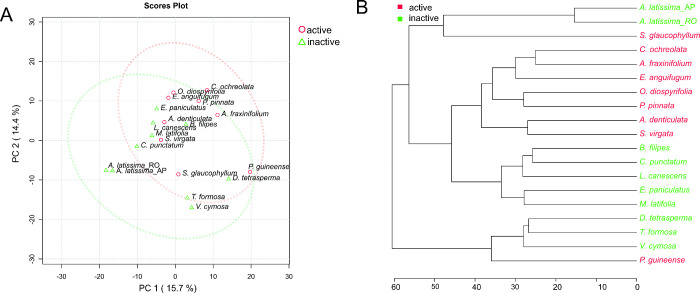
(A) Principal component analysis (PCA) and (B) hierarchical clustering (dendrogram) of the features obtained from LC-MS data of inactive (green) and active (red) extracts.

The PCA explained 5.7 and 14.4% of data variations in PC1 and PC2 (Figure 2A), respectively, showing a tendency for the separation of groups relative to active (red) and inactive extracts (green). From hierarchical clustering, a dendrogram was obtained, and it showed the active extracts from *C. ochreolata*, *A. fraxinifolium*, *E. anguifugum*, *O. diospyrifolia*, *P. pinnata*, *A. denticulata*, and *S. virgata* grouped in the same cluster that revealed some chemical similarities (Figure 2B). Additionally, the active extracts from *P. guineense* and *S. glaucophyllum* were grouped into other clusters.

The heat map from the active and inactive extracts highlighted the chemical differences from the samples, including the common metabolites in different species (Figure S3). The features with statistical significance (*p* < 0.05) and the magnitude of change (fold change) to differentiate the active and inactive extracts were listed from the volcano plot (Figure S4A), and some of their box plots were illustrated (Figure S4B). Thus, the following compounds were highlighted for the active samples: catechin, epicatechin, steroidal saponin (*m*/*z* 741.4426), juribidine (**20**), myricetin, *O*-deoxyhexosyl-hexosyl kaempferol, *O*-deoxyhexosyl kaempferol, *O*-galloyl procyanidin dimer, procyanidin dimer, *O*-pentosyl quercetin, steroidal alkaloid (**17**), and proanthocyanidin (cinnamtannin B_1_) (Figure S4B).

The results also suggested potential bioactive metabolites against leukemia cells, mainly represented by *O*-glycosylated flavonoids, flavan-3-ols, and proanthocyanins. These constituents have been widely reported for several biological activities in the literature. In addition, polyphenols are also described as possible interference of assays since they can act as metal chelators and redox cycling readouts.^14,15^ Thus, a molecular networking strategy was applied to organize the MS and MS/MS data, grouping similar compounds, assisting with the annotation, and improving the determination of the potential active metabolites, such as alkaloids and saponins.

**Figure 3:**
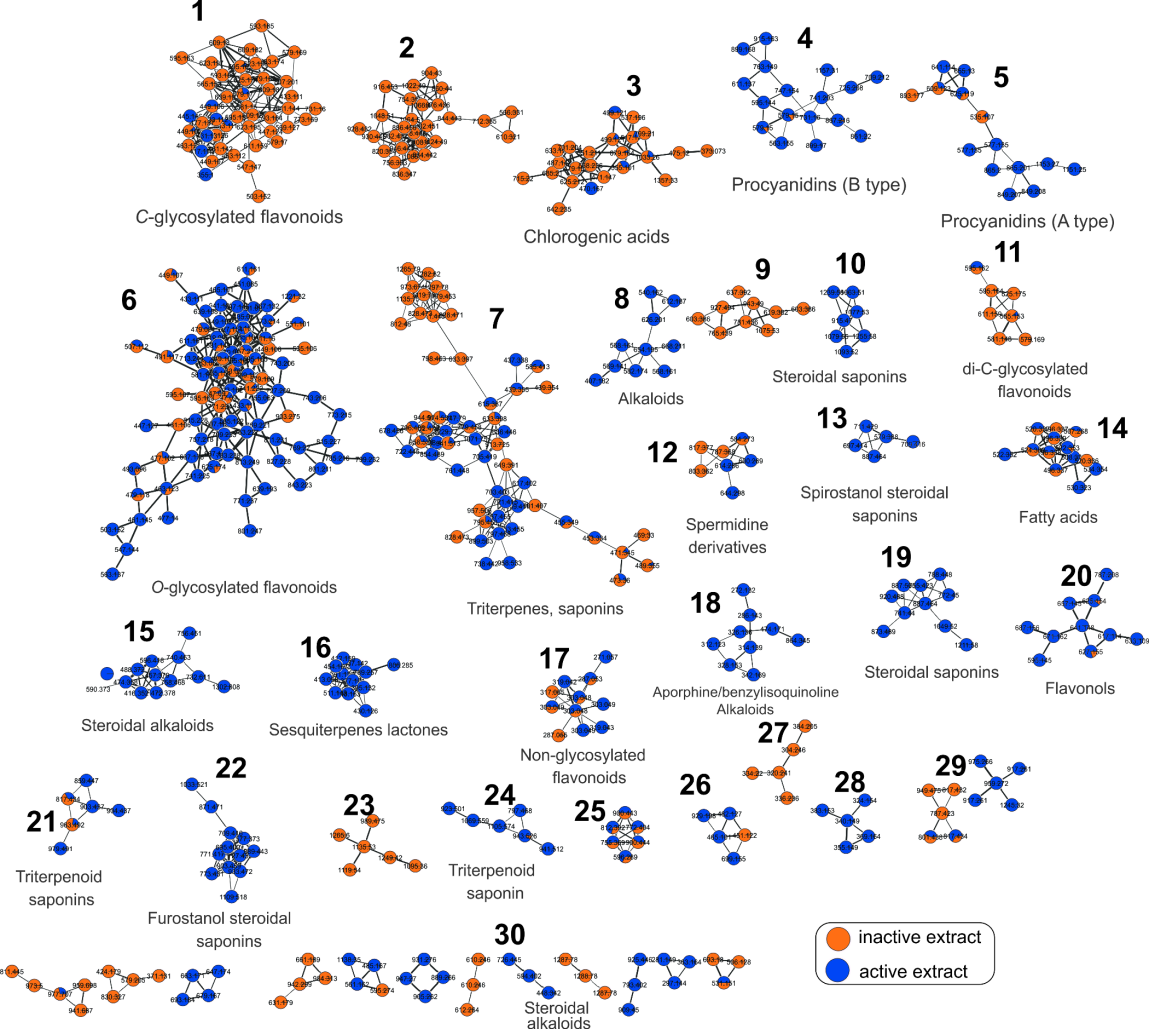
Molecular networking of extracts from all of the plant species. Nodes of nonactive and active samples are colored orange and blue, respectively. The edge strength is proportional to the cosine values.

The blue color features were observed from active extracts in molecular networking, while the orange color features for inactive extracts against leukemia cell lines (Figure 3). The description of the clusters related to metabolite classes is summarized in Table S2 (Supporting Information). They included, mainly, *C*-glycosylated flavonoids (cluster 1), chlorogenic acids (cluster 3), procyanidins (type B-cluster 4 and type A-cluster 5), *O*-glycosylated flavonoids (cluster 6), triterpenes and triterpenoid saponins (clusters 7, 21, and 24), steroidal saponins (clusters 10 and 19), di-*C*-glycosylated flavonoids (cluster 11), spermidine derivatives (cluster 12), spirostanol steroidal saponins (cluster 13), fatty acids (cluster 14), steroidal alkaloids (cluster 15), sesquiterpene lactones (cluster 16), nonglycosylated flavonoids (cluster 17), aporphine alkaloids (cluster 18), *O*-glycosyl-phenylpropanoyl/gallolyl flavonols (cluster 20), and furostanol steroidal saponins (cluster 22).

Features exclusively present in the active samples (blue) were observed in some clusters. These metabolites were extensively studied and annotated since they may represent key compounds with potential activity against leukemia cancer cells. The molecular network categorizes similar features into clusters based on their fragmentation profile, facilitating the selection of target compounds to determine the target compounds. Here, this approach yielded additional insights beyond those obtained from statistical analyses of the chemical data.

**Figure 4:**
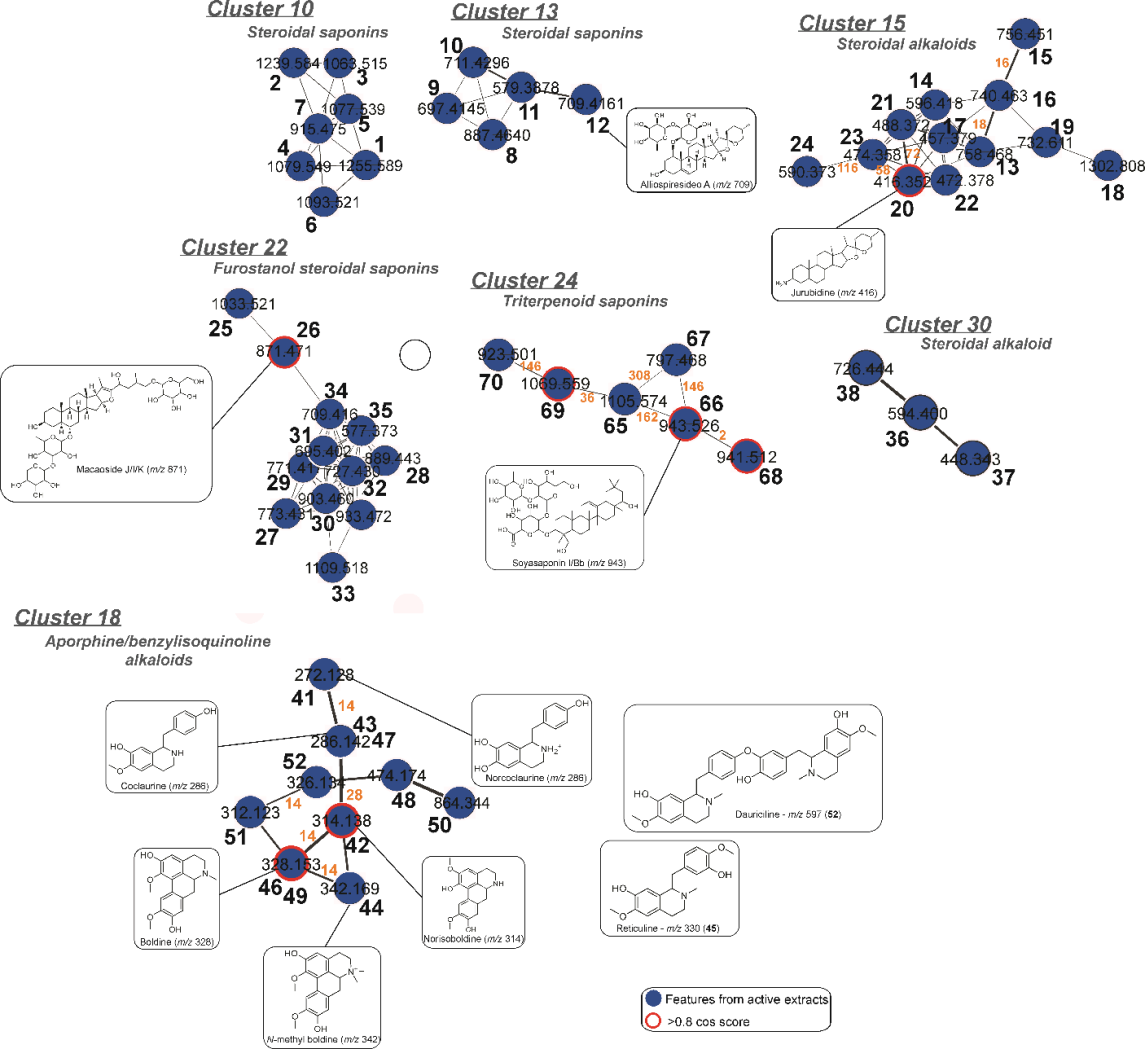
Details of the features from some clusters (10, 13, 15, 18, 22, 24, and 30) and the annotated metabolites detected in *O. diospyrifolia*, *P. pinnata*, and *S. glaucophyllum*.

From cluster 10, features of *S. glaucophyllum* were grouped and they are described in Table 1 and Figure 4. All of the compounds revealed a fragment ion at *m*/*z* 431, which is compatible with C_27_H_42_O_4_ and suggested steroidal aglycones. The metabolites revealed 49, 54, or 55 carbons in their structures. In addition, consecutive losses of sugars from these compounds confirmed steroidal saponins.^16^ Although steroidal saponins are commonly reported from the genus *Solanum*, the saponins grouped in cluster 10 were not found in the literature that suggested new metabolites. Some steroidal saponins from the genus *Solanum* have shown cytotoxic activity against cancer cells that can represent a target group to search for bioactive compounds.^17^

**Table 1:** Compounds Annotated from Clusters 10, 13, 15, 22, and 30 by LC-DAD-MS Data That Were Detected from *Solanum glaucophyllum*^a^

	**RT (min)**	**compound**	**MF**	**MS** (*m*/*z*) **[M + H]**^**+**^	**MS/MS** (*m*/*z*)
Cluster 10					
**1**	30.6	steroidal saponin	C_54_H_96_O_33_	1255.5788^#^	1061, 609, 431, 339, 207
**2**	31.1	steroidal saponin	C_54_H_96_O_32_	1239.5840^#^	909, 593, 431, 339, 287, 207, 175
**3**	32.5	steroidal saponin	C_54_H_80_O_22_	1063.5148^#^	899, 727, 431, 339, 207, 175
**4**	32.6	steroidal saponin	C_55_H_84_O_22_	1079.5493^#^	339, 289, 271, 207
**5**	33.0	steroidal saponin	C_55_H_82_O_22_	1077.5298^#^	431, 339, 287, 207, 175
**6**	33.4	steroidal saponin	C_55_H_80_O_22_	1093.5212	871, 731, 609, 465, 431, 339, 289, 253, 207
**7**	37.9	steroidal saponin	C_49_H_72_O_16_	915.4749	577, 431, 353, 339, 287, 207, 175
Cluster 13					
**8**	40.6	*O*-deoxyhexosyl-hexosyl-pentosyl spirostanol saponin	C_44_H_68_O_18_	887.4640	755, 655, 609, 591, 573, 555, 543, 465, 447, 433, 415, 397, 379, 289, 283, 271, 253
**9**	36.9	di-*O*-pentosyl spirostanol saponin (macaoside D)	C_37_H_60_O_12_	697.4145	565, 433, 385, 289, 271, 253
**10**	37.9	*O*-pentosyl-deoxyhexosyl spirostanol saponin (saponin Sc2)	C_38_H_62_O_12_	711.4296	579, 433, 415, 397, 289, 271, 253
**11**	38.0	*O*-deoxyhexosyl spirostanol saponin (saponin Sc4)	C_33_H_54_O_8_	579.3878	433, 289, 271, 253
**12**	38.2	*O*-deoxyhexosyl-pentosyl spirostanol saponin (alliospiroside A)	C_38_H_60_O_12_	709.4161	577, 431, 413, 395, 287, 269, 251
Cluster 15					
**13**	27.7	di-*O*-glycosylated steroidal alkaloid (*O*-hexosyl jurubine)	C_39_H_67_NO_13_	758.4679	596, 543, 477, 416, 399, 381, 285, 255, 161
**14**	28.0	*O*-glycosylated steroidal alkaloid (jurubine)	C_33_H_57_NO_8_	596.4177	576, 452, 416, 399, 381, 335, 285, 255, 231, 215, 161
**15**	29.8	di-*O*-glycosylated steroidal alkaloid (di-*O*-hexosyl-juripidine)	C_39_H_65_NO_13_	756.4514	739, 676, 596, 524, 490, 474, 432, 416, 415, 399, 397, 379, 284, 271, 253, 229, 215, 159
**16**	29.9	di-*O*-glycosylated steroidal alkaloid (di-*O*-hexosyl juribidine)	C_39_H_65_NO_12_	740.4626	676, 578, 416, 399, 381, 272, 255, 231, 181
**17**	36.1	steroidal alkaloid (unknown)	C_29_H_48_N_2_O_2_	457.3790	416, 399, 381, 353, 285, 273, 255
**18**	37.0	steroidal alkaloid (unknown)	C_64_H_119_NO_25_	1302.8084	1140, 1086, 887, 416, 339, 285, 273, 255
**19**	38.2	steroidal alkaloid (unknown)	C_45_H_81_NO_6_	732.6111	597, 543, 416, 381, 289, 285, 255, 161
**20**	36.1	juribidine*	C_27_H_45_NO_2_	416.3523	399, 381, 285, 255, 235, 215, 161
**21**	36.5	steroidal alkaloid (propionyl-juripidine)	C_30_H_49_NO_4_	488.3722	399, 381, 285, 255, 235, 215, 161
**22**	36.6	steroidal alkaloid (unknown)	C_30_H_49_NO_3_	472.3782	399, 381, 340, 312, 285, 255, 235, 215, 181, 161
**23**	36.8	steroidal alkaloid (acetyl-juripidine)	C_29_H_47_NO_4_	474.3577	441, 416, 399, 381, 298, 285, 273, 255, 231, 187, 161
**24**	39.7	steroidal alkaloid (unknown)	C_33_H_51_NO_8_	590.3730	572, 420, 399, 381, 353, 333, 285, 280, 255, 245, 235, 217, 181, 169, 161
Cluster 22					
**25**	34.8	di-*O*-hexosyl-deoxyhexosyl-pentosyl furostanol saponin	C_50_H_82_O_23_	1033.5208^#^	709, 565, 431, 287, 269
**26**	31.0	*O*-deoxyhexosyl-pentosyl-hexosyl furostanol saponin (macaoside K/J/I)*	C_44_H_72_O_18_	871.4709^#^	739, 709, 577, 541, 431, 413, 395, 287, 269, 251
**27**	33.5	di-*O*-hexosyl furostanol saponin	C_39_H_64_O_15_	773.4315	449, 431, 413, 395, 317, 287, 269, 251, 163
**28**	35.2	furostanol saponin (unknown)	C_43_H_68_O_19_	889.4430	853, 797, 431, 413, 395
**29**	35.3	*O*-deoxyhexosyl-hexosyl furostanol saponin	C_39_H_62_O_15_	771.4173	431, 413, 395, 377, 287, 269
**30**	35.6	*O*-deoxyhexosyl-pentosyl-hexosyl furostanol saponin	C_44_H_70_O_19_	903.4596	741, 625, 463, 431, 413, 395, 317, 305, 299, 287, 269
**31**	37.2	di-*O*-pentosyl furostanol saponin	C_37_H_58_O_12_	695.4002	563, 449, 431, 413, 395, 299, 287, 269, 251
**32**	38.1	*O*-pentosyl-deoxyhexosyl furostanol saponin	C_38_H_62_O_13_	727.4298	595, 449, 431, 413, 395, 377, 287, 269
**33**	37.7	furostanol saponin (unknown)	C_55_H_80_O_23_	1109.5185	449, 431, 413, 395, 339, 305, 287, 207
**34**	38.2	*O*-pentosyl-deoxyhexosyl furostanol saponin (macaoside A)	C_38_H_60_O_12_	709.4161	449, 431, 413, 395, 299, 287, 269, 251
**35**	38.4	*O*-deoxyhexosyl furostanol saponin	C_33_H_52_O_8_	577.3731	431, 413, 395, 317, 299, 287, 269, 251
Cluster 30					
**36**	33.0	*O*-deoxyhexosyl steroidal alkaloid (*O*-deoxyhexosyl esculeogenin A/B)	C_33_H_55_NO_8_	594.3999	448, 430, 412, 395, 286, 269, 251
**37**	33.4	steroidal alkaloid (esculeogenin A or B)	C_27_H_45_NO_4_	448.3432	430, 412, 286, 251, 239
**38**	33.7	*O*-deoxyhexosyl-pentosyl steroidal alkaloid (*O*-deoxyhexosyl-pentosyl esculeogenin A/B)	C_38_H_63_NO_12_	726.4440	594, 576, 558, 540, 490, 472, 448, 430, 412
Other compounds					
**39**	23.5	*N*-*trans*-coumaroyl tyramine	C_17_H_17_NO_3_	284.1299	147
**40**	25.0	*N*-*trans-*feruloyl tyramine	C_18_H_19_NO_4_	314.1401	177

a RT: retention time; MF: molecular formula. The MF was considered with errors up to 8 ppm and mSigma 30. *>0.8 cos score; ^#^[M + H–H_2_O]^+^.

The metabolites grouped in cluster 13 did not exhibit UV absorption, and they revealed consecutive losses of sugars such as 176, 162, 146, and 132 *u*, indicating the presence of glucuronyl, hexosyl, deoxyhexosyl, and pentosyl substituents, respectively.^16,18^ These data were similar to those of the metabolites grouped in cluster 22 (Table 1). The aglycones of features from clusters 13 and 22 exhibited the ions at *m*/*z* 433 (C_27_H_44_O_4_) and 431 (C_27_H_42_O_4_), and the consecutive losses of water molecules (18 *u*) from them were observed such as the ions *m*/*z* 415, 397, 413, and 395, which are compatible with losses observed for steroidal aglycone. The losses of 144 *u*, yielded by cleavage from the E-ring of steroidal aglycone with subsequent losses of the water molecule, confirmed spirostanol saponin (metabolites from cluster 13), and, additionally, the furostanol saponins (metabolites from cluster 22) were suggested when the losses of 114 *u* were also observed.^16,18,19^ For example, fragment ions *m*/*z* 289 [aglycone(433)+H-144]^+^, 271 [aglycone(433)+H-144-H_2_O]^+^, and 253 [aglycone(433)+H-144-2xH_2_O]^+^ were observed for **8**–**11**. Thus, it was possible to annotate several spirostanol (**8**–**12**) and furostanol steroidal saponins (**25**–**35**) and they were detected only from the extract of *S. glaucophyllum*, which was active mainly against K-562 leukemia cancer cells.

The saponin **26**, a furostanol saponin, was putatively annotated as macaoside K/J or I, which has demonstrated an EC_50_ of 14.2–37.6 μg/mL against SK-Lu-1, HepG2, MCF-7, and T24 cancer cell lines.^20^ These results highlight potential targets to research new compounds against cancer cells since some saponins from this cluster were not evaluated for them or reported in the literature yet, such as **31**, **33**, and **35**.

The metabolites **13**–**24** (cluster 15) did not exhibit UV absorption, presented nitrogen in their molecular formulas, and revealed losses of glycosides (e.g., hexosyl 162 *u*) that indicated steroidal alkaloids, a common metabolite class reported for *Solanum* species.^17^ In MS/MS, the aminospirane alkaloids lose an amine (17 *u*) and the spirostane ring by sequential dehydration and hydrogen rearrangement, which occur by the ring opening with anchimeric assistance, to yield the diagnostic fragment ions.^21^ The metabolite **13** (*m*/*z* 758.4679 [M + H]^+^), for example, yielded the product ions by losses of hexosyl groups (*m*/*z* 596 [M+H-162]^+^ and 416 [M+H-2x162-H_2_O]^+^), amine (*m*/*z* 399 [416+H-NH_3_]^+^), and the losses relative to the spirostane ring (*m*/*z* 285 and 255). Thus, alkaloid **13** was annotated as *O*-hexosyl jurubine (di-*O*-glycosylated steroidal alkaloid).

The steroidal alkaloids of cluster 15 (Figure 4) presented similar fragmentation pathways, and the molecular network also showed these similarities and the mass differences of features. Compound **23** showed a mass difference relative to an acetyl group (−OCOCH_3_) compared to metabolite **20** and was annotated as acetyl-juripidine. Additionally, other glycosylated steroidal alkaloids were also annotated, such as jurubine (*O*-glycosyl steroidal alkaloid) (**14**), di-*O*-hexosyl-juripidine (**15**), di-*O*-hexosyl juribidine (**16**), juribidine (**20**), propionyl-juripidine (**21**), and other unknown steroidal alkaloids (**17***m*/*z* 457.3790, **18***m*/*z* 1302.8084, **19***m*/*z* 732.6111, **22***m*/*z* 472.3782, and **24***m*/*z* 590.3730). The alkaloids from cluster 15 are scarcely studied, but they are an excellent aim to search new cytotoxic compounds since there are several cytotoxic data reported for the metabolite class of the steroidal alkaloids of *Solanum* species.^17^

The cluster 18 is composed of benzylisoquinoline and aporphine alkaloids (Figure 4), and the fragmentation pathway of them was similar, such as the losses of 17 (NH_3_), 30 (CH_2_O), and 15 *u* (^·^CH_3_), which confirmed the absence of a methyl group in the nitrogen, the methylenedioxy, and methoxyl substituents (Figure S5). For example, the alkaloid **51** showed an intense ion at *m*/*z* 312.1234 [M + H]^+^ compatible to the molecular formula C_18_H_14_NO_4_. Its fragment ions were observed at *m*/*z* 295 [M+H-NH_3_]^+^, 280 [M+H-NH_3_-^·^CH_3_]^+^, 265 [M+H-NH_3_-CH_2_O]^+^, and 237 [M+H-NH_3_-CH_2_O-CO]^+^ (Table 2). Thus, the alkaloid **51** was annotated as hernangerine/litsferine and the spectral data were compatible with those reported for it.^22^ In addition, the alkaloid boldine (**46**) was also annotated, which showed a hit with the spectral data deposited in GNPS and was confirmed by injection of an authentic standard. All the alkaloids grouped in cluster 18 are summarized in Table 2 and also included the annotated alkaloids norcoclaurine (**41**), norisoboldine (**42**), and *N*-methyl boldine (**44**).

**Table 2:** Alkaloids Annotated from *O. diospyrifolia* and *P. pinnata* by LC-DAD-MS Data^a^

**no.**	**RT (min)**	**compound**	**UV (nm)**	**MF**	**[M + H]**^**+**^	**MS/MS**	**sample**
**41**	8.5	norcoclaurine	280	C_16_H_17_NO_3_	272.1321	255, 237, 209, 194, 165	*O. diospyrifolia*
**42**	11.4	norisoboldine*	277, 310 (sh)	C_18_H_19_NO_4_	314.1390	297, 282, 265, 177	*O. diospyrifolia, P. pinnata*
**43**	13.0	coclaurine	282, 310 (sh)	C_17_H_19_NO_3_	286.1440	269, 254, 237, 219, 209, 194, 191, 178, 175, 166	*O. diospyrifolia*
**44**	14.8	*N*-methyl boldine	280, 310 (sh)	C_20_H_23_NO_4_	342.1695	297, 282, 265, 251, 237, 222, 189, 165	*O. diospyrifolia*
**45**	15.3	reticuline	279, 330 (sh)	C_19_H_23_NO_4_	330.1694	299, 267, 192, 177	*O. diospyrifolia*
**46**	16.4	boldine*	279, 309 (sh)	C_19_H_21_NO_4_	328.1535	297, 282, 265, 250, 237, 233, 205	*O. diospyrifolia, P. pinnata*
**47**	17.3	coclaurine isomer	279, 309 (sh)	C_17_H_19_NO_3_	286.1434	269, 254, 237, 219, 209, 194, 178, 175, 165, 163	*O. diospyrifolia*
**48**	18.1	alkaloid (unknown)	276	C_31_H_23_NO_4_	474.1710	456, 438, 295, 265	*O. diospyrifolia*
**49**	18.9	isoboldine	282, 307	C_19_H_21_NO_4_	328.1543	297, 282, 265, 253, 237, 207	*O. diospyrifolia*
**50**	19.0	alkaloid	278, 307	C_45_H_53_NO_16_	864.3447	591, 455, 312, 195, 165	*O. diospyrifolia*
**51**	18.8	hernangerine/litsferine	279, 312 (sh)	C_18_H_17_NO_4_	312.1234	295, 280, 265, 250, 237, 222, 205	*O. diospyrifolia, P. pinnata*
**52**	19.0	*N*-methylhernangerine/*N*-methylactinodaphine/domesticine	282, 307	C_19_H_19_NO_4_	326.1361	311, 295, 280, 265, 250, 237, 222, 205	*O. diospyrifolia*
**53**	20.3	dauriciline	282	C_36_H_40_N_2_O_6_	597.2958	192, 178	*O. diospyrifolia*

a RT: retention time; MF: molecular formula. The MF was considered with errors up to 8 ppm and mSigma 30. *>0.8 cos score in GNPS and MZmine.

The alkaloid **51**, hernangerine/litsferine, is an aporphinoid alkaloid with a 1,2-methylenedioxy, and this group has been related to better cytotoxic activities since there is better stability of interaction in relation to thermal denaturation of DNA double helices compared to aporphinoids without a 1,2-methylenedioxy group; in addition, they promoted inhibition of topisomerase I by intercalation into DNA that can explain the cytotoxic properties.^23^ Therefore, these alkaloids could be good targets against leukemia and cancer cells.

Cluster 20 (Figure S6) is mainly composed of features of active samples, and these metabolites are annotated and summarized in Table S3. This cluster is yielded from flavonols linked to glycosides and phenylpropanoids (sinapoyl, feruloyl, or coumaroyl) or galloyl substituents. The aglycones annotated in this cluster were myricetin, quercetin, and kaempferol. Therefore, the annotated flavonols from cluster 20 were the following: *O*-galloyl-hexosyl myricetin (*m*/*z* 633.1090 [M + H]^+^) (**54**), *O*-galloyl-hexosyl quercetin (*m*/*z* 617.1141) (**55**), di-*O*-hexosyl quercetin (*m*/*z* 627.1546) (**56**), *O*-sinapoyl-hexosyl myricetin (*m*/*z* 687.1564) (**57**), *O*-feruloyl-hexosyl myricetin (*m*/*z* 657.1448) (**58**), *O*-feruloyl dihexosyl kaempferol (*m*/*z* 787.2078) (**59**), *O*-sinapoyl-hexosyl quercetin (*m*/*z* 671.1621) (**60**), *O*-feruloyl-hexosyl quercetin (*m*/*z* 641.1481) (**61, 62**), *O*-coumaroyl-hexosyl kaempferol (*m*/*z* 595.1446) (**63**), and *O*-feruloyl-hexosyl kaempferol (*m*/*z* 625.1538) (**64**). Additionally, the compounds from cluster 28 are described in Table S4, and the annotation performed by the comparison of the mass spectra library in MZmine is summarized in Table S5 (Supporting Information).

The metabolites **65**–**70** were grouped in cluster 24, which are present only in the *A. denticulata* extract (Figure 4 and Table 3). These constituents did not show UV absorption, and their molecular formulas, determined from the accurate MS, revealed 42, 48, or 54 carbons. The fragment ions from aglycones (*m*/*z* 441 and 439 [aglycone+H-H_2_O]^+^ for C_30_H_48_O_2_ and C_30_H_46_O_2_, respectively) and the losses of glycosides (hexosyl 162 *u*, deoxyhexosyl 146 *u*, and glucuronyl 176 *u*) confirmed triterpenoid saponins; in addition, losses of water molecules were commonly observed, as cited in the literature.^24^ The mass differences between the saponins in cluster 24 were highly relevant for the annotation of the saponins, such as for compounds **65** (*m*/*z* 1105.5741) and **66** (*m*/*z* 943.5265) that presented a difference of a hexosyl (162 *u*). Thus, the annotated saponins were di-*O*-hexosyl deoxyhexosyl glucuronyl triterpenoid saponin (abrisaponin SB or D2) (**65**), *O*-hexosyl deoxyhexosyl glucuronyl triterpenoid saponin (soyasaponin I or Bb) (**66**), *O*-hexosyl glucuronyl triterpenoid saponin (soyasaponin III or Bb’) (**67**), *O*-hexosyl deoxyhexosyl glucuronyl triterpenoid saponin (dehydrosoyasaponin I) (**68**), di-*O*-hexosyl deoxyhexosyl glucuronyl triterpenoid saponin (soyasaponin VI or βg) (**69**), and di-*O*-hexosyl glucuronyl triterpenoid saponin (soyasaponin γg) (**70**).

**Table 3:** Compounds Annotated from Cluster 24 by LC-DAD-MS Data That Were Detected Only from *Aeschynomene denticulata*^a^

	**RT (min)**	**compound**	**MF**	**[M + H]**^**+**^	**MS/MS** (*m*/*z*)
**65**	30.4	di-*O*-hexosyl deoxyhexosyl glucuronyl triterpenoid saponin (abrisaponin SB/D2)	C_54_H_88_O_23_	1105.5741	581, 441, 423, 405, 365, 217
**66**	34.0	*O*-hexosyl deoxyhexosyl glucuronyl triterpenoid saponin (soyasaponin I/Bb)*	C_48_H_78_O_18_	943.5265	797, 599, 441, 423, 405
**67**	34.3	*O*-hexosyl glucuronyl triterpenoid saponin (soyasaponin III/Bb’)	C_42_H_68_O_14_	797.4676	639, 599, 581, 441, 423, 405, 365, 201,
**68**	34.6	*O*-hexosyl deoxyhexosyl glucuronyl triterpenoid saponin (dehydrosoyasaponin I)*	C_48_H_76_O_18_	941.5124	597, 439, 421, 313, 245
**69**	35.03	di-*O*-hexosyl deoxyhexosyl glucuronyl triterpenoid saponin (soyasaponin VI/βg)*	C_54_H_84_O_21_	1069.5589	923, 743, 599, 441, 423, 405, 217, 127
**70**	35.4	di-*O*-hexosyl glucuronyl triterpenoid saponin (soyasaponin γg)	C_48_H_74_O_17_	923.5008	599, 581, 567, 549, 549, 441, 423, 405, 365, 297, 217

a RT: retention time; MF: molecular formula. The MF was considered with errors up to 8 ppm and mSigma 30. *>0.8 cos score.

### 3.3 Cytotoxicity Effects of Alkaloids from *S. glaucophyllum* and *O. diospyrifolia* and Flow Cytometry

**Figure 5:**
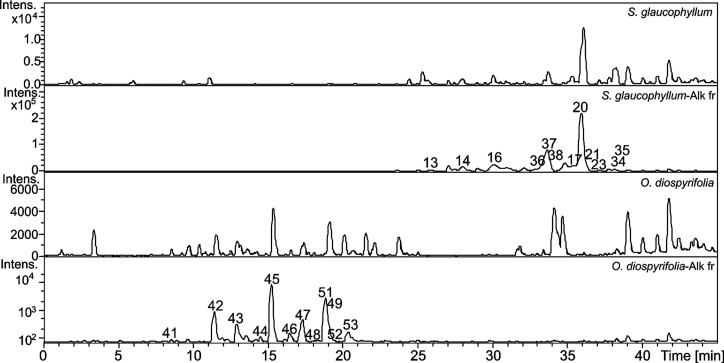
Base peak chromatograms from *S. glaucophyllum* and *O. diospyrifolia* extracts and their alkaloid fractions (Alk_fr).

The extracts from *S. glaucophyllum* and *O. diospyrifolia* were subjected to alkali-acid extraction, yielding their respective alkaloid fractions (Alk_fr). These fractions were analyzed by LC-DAD-MS, and their chromatogram profiles are illustrated in Figure 5. In the Alk_fr of *S. glaucophyllum*, alkaloids **13**–**14**, **16**–**17**, **20**–**21**, **23**, and **34**–**38** were annotated, while alkaloids **41**–**49** and **51**–**53** were detected in the Alk_fr of *O. diospyrifolia*. These classes of alkaloids have shown cytotoxic properties against cancer cell lines, representing an important source for drug discovery.^25,26^

**Figure 6:**
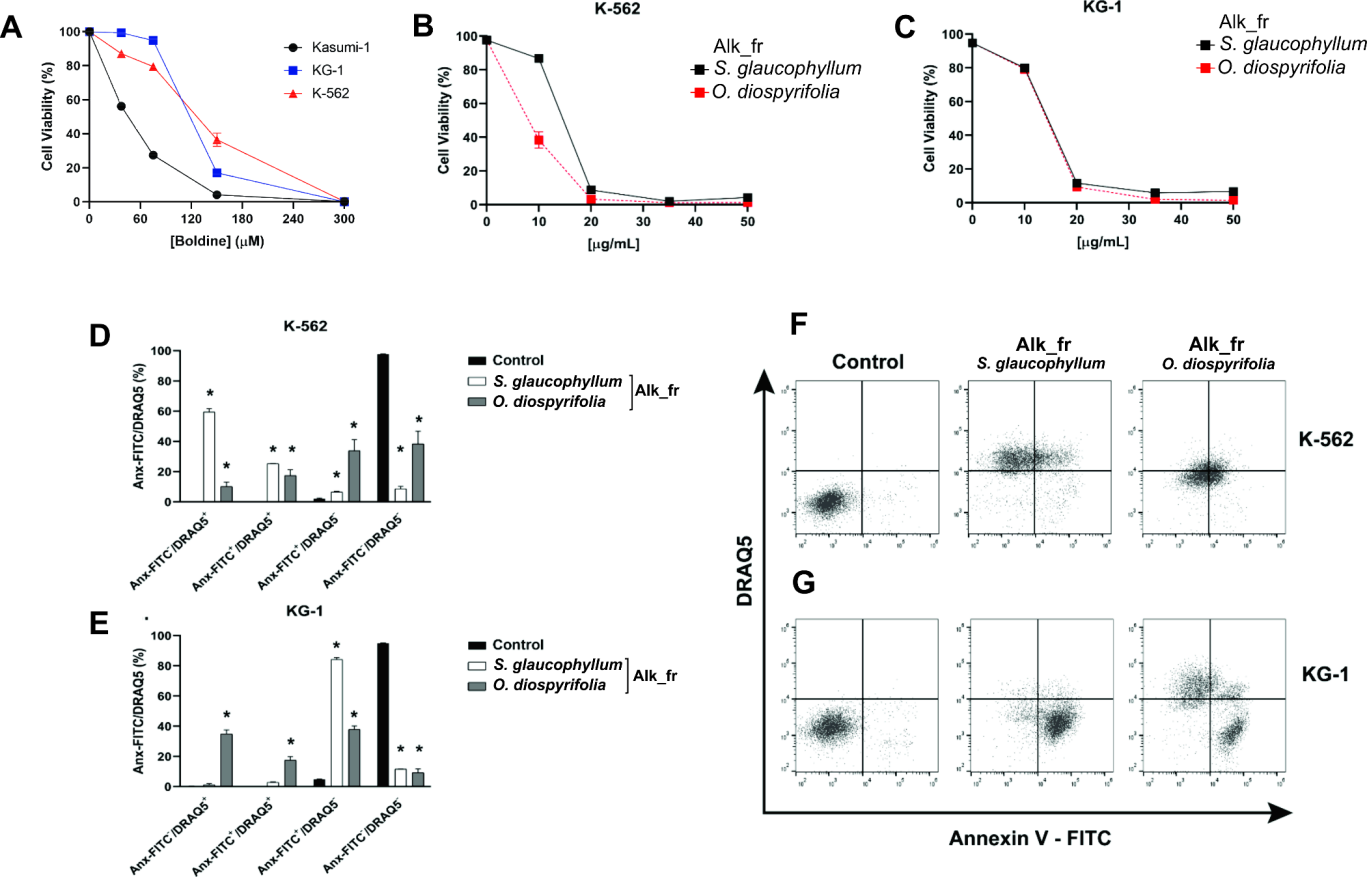
Cell proliferation and cytotoxic profile of boldine on Kasumi-1, K-562, and KG-1 cells (A) and alkaloid fractions (Alk_fr) from *S. glaucophyllum* and *O. diospyrifolia* on K-562 (B) and KG-1 (C). Cell viability was assessed by using a resazurin assay. The cytotoxic activities of Alk_fr from *S. glaucophyllum* and *O. diospyrifolia* (D, E) in K-562 and KG-1 were assessed by analysis of Annexin V-FITC/DRAQ5 staining after 24 h with EC_50_ for 24 h (F, G). The results are the means ± SEM of three independent experiments performed in triplicate. *P < 0.05. One-way ANOVA followed by Dunnett’s post hoc test was used.

The K-562 cell line demonstrates the highest sensitivity among the leukemia lines evaluated in this study, whereas the KG-1 line showed greater resistance.^27^ These two cell lines were therefore selected to assess the cytotoxic activity of the alkaloid fractions from *S. glaucophyllum* and *O. diospyrifolia* (Figure 6), and EC_50_ values were calculated (Table 4). For the K-562 lineage, the EC_50_ values of alkaloid fractions from *S. glaucophyllum* and *O. diospyrifolia* were 13.9 and 6.4 μg/mL, respectively, while for the KG-1 lineage, the values were 13.5 and 13.4 μg/mL. The alkaloid fractions exhibited higher cytotoxic activity compared to the crude extracts of *S. glaucophyllum* and *O. diospyrifolia*, suggesting that these alkaloids can contribute to the antileukemic activity observed.

**Table 4:** EC_50_ of Alkaloid Fractions (Alk_fr) from *S. glaucophyllum* and *O. diospyrifolia* and Boldine against Leukemia Cancer Cell Lines^a^

	**cell line (μg/mL)**		
**sample**	**K-562**	**KG-1**	**Kasumi-1**
Alk_fr *S. glaucophyllum*	13.9	13.5	N.D.
Alk_fr *O. diospyrifolia*	6.4	13.4	N.D.
boldine	145 μM	116 μM	46 μM

a N.D.: not determined.

Boldine (**46**), a natural aporphine alkaloid, was evaluated at different concentrations against Kasumi-1, KG-1, and K-562 leukemia cells (Figure 6A–C) and revealed EC_50_ of 46, 116, and 145 μM, respectively (Table 4). This alkaloid has been evaluated against various cancer cell lines, showing EC_50_ values of 46.5, 50.8, and 160 μg/mL for MDA-MB231, MDA-MB468, and MCF-7 human breast cancer cells.^26,28^ Additionally, boldine has been reported to induce the activation of caspase-9 and caspase-3/7 and inhibits the kappa B factor and breast cancer in vivo at doses of 50 or 100 mg/kg in animal models. In addition, this alkaloid did not demonstrate acute toxicity at a dose of 100 mg/kg,^28^ and it showed activity against U138-MG human glioma cells and increased the cells in the G2/M phase after 24 h.^25^

The alkaloids norisoboldine (**42**), reticuline (**45**), and hernangerine/litsferine (**51**) were the most intense of the peaks in Alk_fr of *O. diospyrifolia*. Hernangerine (**51**) has shown EC_50_ of 1.09 (3.5 μM), 1.53, 2.83, and 2.94 μg/mL against P388 leukemia, KB-16 nasopharyngeal, A549 nonsmall cell lung, and HT29 colorectal human cancer cell lines, respectively.^29^ Reticuline has revealed EC_50_ of 38, 32.1, and 18.3 μM for SUP-B15 (acute lymphoblastic leukemia), KOPN-8 (acute lymphoblastic leukemia with MLL-MLLt1/ENL fusion), and NALM-06 (non-T/non-B acute lymphoblastic leukemia at relapse with P15INK4B and P16INK4A deletions), respectively.^30^

The cytotoxic activity induced by alkaloid fractions (Alk_fr) of *S. glaucophyllum* and *O. diospyrifolia* was investigated through Annexin V and DRAQ5 staining to evaluate the treatments for phosphatidylserine externalization and membrane integrity loss. An Anx+/DRAQ5- response indicates phosphatidylserine externalization and the onset of early apoptosis. In contrast, double staining (Anx+/DRAQ5+) indicates late apoptosis/necrosis-like characterized by both the loss of membrane integrity and labeling of phosphatidylserine.^31^ The treatment with the EC_50_ of the alkaloid fraction of *S. glaucophyllum* on K-562 (Figure 6D,F) resulted in a significant reduction in cell viability, predominantly due to compromised plasma membrane integrity. In contrast, the treatment with an alkaloid fraction of *O. diospyrifolia* at a concentration of EC_50_ also reduced the cell viability, but it showed relatively less impact on plasma membrane integrity. For KG-1 cells (Figure 6D,G), the exposure to the alkaloid fraction of *S. glaucophyllum* induced a milder reduction in membrane integrity with cell death displaying apoptotic characteristics, while the alkaloid fraction of *O. diospyrifolia* was associated with the loss of membrane integrity and the onset of late apoptosis.

**Figure 7:**
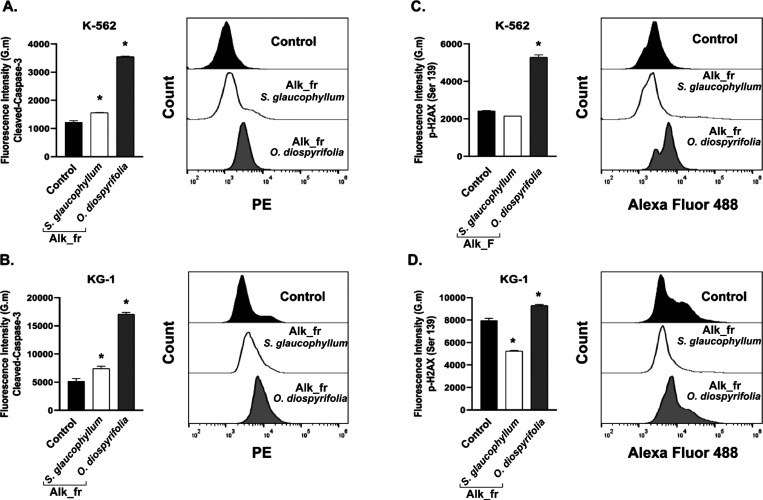
Analysis of apoptosis and DNA damage cell signaling from the alkaloid fractions (Alk_fr) of *S. glaucophyllum* and *O. diospyrifolia*. (A, B) Cleaved caspase-3 activation. Activation of p-H2AX (Ser-139) in K-562 (C) and KG-1 cells after 24 h (D) and typical flow cytometry histograms. The results are the means ± SEM of three independent experiments performed in triplicate. *P < 0.05. One-way ANOVA followed by Dunnett’s post hoc test was used.

Evidence that alkaloid fractions of *S. glaucophyllum* and *O. diospyrifolia* induced apoptosis in K-562 and KG-1 cells prompted an assessment of whether these treatments effectively triggered caspase-3 activation. In both K-562 and KG-1 cells, the treatments increased cleaved caspase-3 and the alkaloid fraction of *O. diospyrifolia* induced approximately a 3-fold increase in both cell lines (Figure 7A,B). Caspases are endopeptidases that cleave specific sites on approximately 1500 substrates and play a crucial role in mediating apoptosis. Among them, caspase-3 is considered the most critical enzyme in this process. The activation of caspase-3 is commonly linked with phosphatidylserine externalization and other morphological changes characteristic of apoptosis.^32^ Additionally, the phosphorylation of the Ser-139 residue in the H2AX histone variant constitutes an early cellular response to the induction of double-strand breaks in DNA. This phosphorylation is regarded as a specific and sensitive molecular marker of DNA damage.^33^ In K-562 and KG-1 cells, a significant increase in p-H2AX was observed only for the treatment with the Alk_fr of *O. diospyrifolia*, whereas in KG-1 cells, the Alk_fr of *S. glaucophyllum* caused a decrease in phosphorylation in this protein.

Therefore, our findings are significant and highlight potential antileukemic extracts and lead compounds, such as aporphine alkaloids. It is important to note that the inclusion of diverse plant families with various chemical constituents posed challenges for statistical analysis. However, these analyses were conducted thoroughly and complemented by molecular networking, which assisted in the determination of lead compounds. Furthermore, the most representative samples identified in this study can serve as a source for future research focusing on specific plant genera or families, mainly involved in alkaloid biosynthesis. In addition to the active extracts from *O. diospyrifolia* and *S. glaucophyllum* that demonstrated efficacy against leukemia cell lines, our study also identified other promising samples. These samples have potential for future research aimed at discovering bioactive metabolites and elucidating their mechanisms of action.

## 4 Conclusions

Our study highlights the innovative application of metabolomics and molecular networking to identify potential bioactive compounds against leukemia cancer cells. By the integration of advanced statistical analyses and targeted metabolite annotation, we successfully distinguished chemical differences across plant extracts from 14 families. Then, steroidal and aporphine alkaloids were identified as promising candidates for cytotoxic activity, particularly those derived from *Solanum glaucophyllum* and *Ocotea diospyrifolia*, which demonstrated significant proapoptotic effects, caspase-3 activation, and DNA damage induction.

The alkaloid fractions exhibited superior cytotoxic activity compared to that of crude extracts, emphasizing the importance of alkaloids for the activity. Furthermore, the approach of molecular networking enabled the discovery of unique metabolite clusters, including novel steroidal saponins and aporphine alkaloids, which represent valuable leads for antileukemic drug development. Therefore, these strategies employed in this study serve as a connection between chemical diversity and targeted bioactivity, providing a robust framework for future investigations of natural products as potential therapeutic agents.

## Supporting Information Available

(Table S1) Plant species, (Tables S2–S5) spectral data and annotation of the compounds from the selected clusters, (Figure S1) cytotoxic activities of the extracts on leukemia cell lines at 10 μg/mL, (Figure S2) base peak chromatograms from the extracts, (Figures S3 and S4) multivariate statistical analysis, (Figure S5) fragmentation pathway of the metabolites, and (Figure S6) clusters of the molecular networking from the chemical data (PDF)
