# Supplementary material for: Deciphering Steroidal and Aporphine Alkaloids as Antileukemic Agents by Approaches of Molecular Networking and Metabolomics
Source: ACS Omega. 2025 Mar 6;10(10):10327–39. doi: 10.1021/acsomega.4c10160 (PMC11923848; doi:10.1021/acsomega.4c10160)
Supplement: Supplementary file 1 — ao4c10160_si_001.pdf [file ao4c10160_si_001.pdf]

SUPPORTING INFORMATION

Deciphering Steroidal and Aporphine Alkaloids as Anti-Leukemic Agents by approaches of Molecular Networking and Metabolomics

Suni Liu[1], Katyuce Souza Farias[1], Vanessa Samudio Santos Zanuncio[1], Geraldo Alves Damasceno Júnior[2], Flávio Macedo Alves[2], Edgar J Paredes-Gamero[3], Kamylla Fernanda Souza de Souza[3,4], Lucas Roberto Pessatto[3], Heron Fernandes Vieira Torquato[3], Carlos Alexandre Carollo[1], Denise Brentan Silva[1,*]

[1]Federal University of Mato Grosso do Sul, Faculty of Pharmaceutical Sciences, Food and Nutrition (FACFAN), Laboratory of Natural Products and Mass Spectrometry (LaPNEM), 79070-900, Campo Grande, Mato Grosso do Sul, Brazil.

[2]Federal University of Mato Grosso do Sul, Laboratory of Botany, Institute of Biosciences (INBIO), 79070-900, Campo Grande, Mato Grosso do Sul, Brazil.

[3]Federal University of Mato Grosso do Sul, Laboratory of Molecular Biology and Cell Cultures, Faculty of Pharmaceutical Sciences, Food and Nutrition (FACFAN), 79070-900, Campo Grande, Mato Grosso do Sul, Brazil.

[4]Universidade Federal de São Paulo, Biochemistry Department, 04044-020, São Paulo, SP , Brazil.

*Corresponding author: D. B. Silva, Laboratory of Natural Products and Mass Spectrometry, Faculty of Pharmaceutical Sciences, Food and Nutrition (FACFAN), Federal University of Mato Grosso do Sul, Av. Costa e Silva, s/nº, 79070-900, Campo Grande, Mato Grosso do Sul, Brazil. Tel.: +55 (67) 3345-7366.

Email: denise.brentan@ufms.br (D.B. Silva).

Summary

Tables

Table S1. Description of species used in the study, botanic family, the used part of the plants, and the number of vouchers deposited in herbarium CGMS of Federal University of Mato Grosso do Sul.

Table S2. Description of the metabolite classes grouped in the molecular networking of figure 1.

Table S3. Compounds annotated from cluster 20 by LC-DAD-MS data.

Table S4. Compounds annotated from cluster 28 by LC-DAD-MS data.

Table S5. Compounds annotated by MZmine from LC-MS/MS data.

Figures

Figure S1. Cell proliferation and cytotoxic screening of the polar extracts on K562 (A), Kasumi-1 (B), and KG-1 (C) leukemia cell lines at 10µg/mL for 24h. Extracts from the following species*:* *Sesbania virgata* (1), *Cenetrratherum punctatum* (2), *Lantana canescens* (3), *Melanthera latifolia* (4), *Aeschynomene denticulata* (5), *Echinodorus paniculatus* (6), *Byttemeria filipes* (7), *Aspilia latissima* – aerial parts (AP) (8), *A. latissima* - roots (9), *Erythroxylum anguifugum* (10), *Tocoyena formosa* (11), *Diospyros tetrasperma* (12), *Psidium guineense* (13), *Vitex cymosa* (14), *Astronium fraxinifolium* (15), *Coccoloba ochreolata* (16), *Solanum glaucophyllum* (17), *Paullinia pinnata* (18), and *Ocotea diospyrifolia* (19). were evaluated. Doxorubicin was used as positive control. Results were expressed as mean ± standard deviation (SD).

Figure S2. Base peak chromatogram obtained in positive ion mode from *Sesbania virgata*, *Cenetrratherum punctatu*, *Lantana canescens*, *Melanthera latifolia*, *Aeschynomene denticulata*, *Echinodorus paniculatus*, *Byttemeria filipes*, *Aspilia latissima* – aerial parts, *A. latissima* - roots, *Erythroxylum anguifugum*, *Tocoyena formosa*, *Diospyros tetrasperma*, *Psidium guineense*, *Vitex cymosa*, *Astronium fraxinifolium*, *Coccoloba ochreolata*, *Solanum glaucophyllum*, *Paullinia pinnata*, and *Ocotea diospyrifolia*.

Figure S3. Heatmap and hierarchical clustering (HCA) of ion intensities of the top 100 features. The samples were classified in the groups active (red) and inactive (green) against leukemia cancer cells. AP: aerial parts; RO: roots.

Figure S4. Volcano plot displaying the differences in features between the active (right quadrant) and inactive extracts (left quadrant). The x and y axes represent the fold change (FC) and P values of the features highlighted showed $p \leq 0.05$ and they were illustrated in the box plots below (A). Box plots from the annotated features that revealed $p \leq 0.05$ for the active samples (B).

Figure S5. Fragmentation pathway for tyramine derivatives (A) and aporphine alkaloids (B).

Figure S6. Cluster of *O*-glycosyl-phenylpropanoyl/gallolyl flavonols (cluster 20). The annotation of features is described in Table S3.

Parameters applied for data Processing by MZmine

Table S1. Description of species used in the study, botanic family, the used part of the plants, and the number of vouchers deposited in herbarium CGMS of Federal University of Mato Grosso do Sul.

Plant species name	Family	Part of the plant	Voucher
Sesbania virgata (Cav.) Pers.	Fabaceae	AP/FR	CGMS 34909
Centratherum punctatum Cass.	Asteraceae	AP/FL	CGMS 34910
Lantana canescens Kunth	Verbenaceae	AP/FL	CGMS 34911
Melanthera latifolia (Gardner) Cabrera	Asteraceae	AP/FL	CGMS 34913
Aeschynomene denticulata Rudd	Fabaceae	AP/FL/FR	CGMS 34916
Echinodorus paniculatus Micheli	Alismataceae	AP/FL	CGMS 34917
Byttneria filipes Mart. ex K.Schum.	Malvaceae	AP/FL	CGMS 34925
Aspilia latissima Malme	Asteraceae	AP	CGMS 34914
Aspilia latissima Malme	Asteraceae	RO	CGMS 34914
Erythroxylum anguifugum Mart.	Erythroxylaceae	AP	CGMS 34948
Tocoyena formosa (Cham. & Schltdl.) K.Schum.	Rubiaceae	AP	CGMS 34946
Diospyros tetrasperma Sw.	Ebenaceae	AP	CGMS 34944
Psidium guineense Sw.	Myrtaceae	AP/FR	CGMS 34942
Vitex cymosa Bertero ex Spreng.	Lamiaceae	AP	CGMS 34940
Astronium fraxinifolium Schott	Anacardiaceae	AP	CGMS 34939
Coccoloba ochreolata Wedd.	Fabaceae	AP	CGMS 34937
Solanum glaucophyllum Desf.	Solanaceae	FR	CGMS 34951
Paullinia pinnata L.	Sapindaceae	AP/FR	CGMS 34953
Ocotea diospyrifolia (Meisn.) Mez	Lauraceae	AP	CGMS 34954

AP: aerial parts; LV: leaves; RO: Root, FL: Flower; FR: fruit

Table S2. Description of the metabolite classes grouped in the molecular networking of figure 1.

Cluster	Metabolite Class	Cluster	Metabolite Class
**1**	*C*-glycosylated flavonoids	**16**	Sesquiterpene lactones
**2**	Unknown	**17**	Non-glycosylated Flavonoids
**3**	Chlorogenic acids	**18**	Aporphine alkaloids
**4**	Procyanidins – type B	**19**	Steroidal saponins
**5**	Procyanidins – type A	**20**	*O*-glycosyl-phenylpropanoyl/gallolyl flavonols
**6**	*O*-glycosytaled flavonoids	**21**	Triterpenoid saponins
**7**	Triterpenes and triterpenoid saponins	**22**	Furostanol steroidal saponins
**8**	Alkaloid derivatives	**23**	Unknown
**9**	Unknown	**24**	Triterpenoid saponin
**10**	Steroidal saponins	**25**	Unknown
**11**	di-*C*-glycosylated flavonoids	**26**	Flavonoids
**12**	Spermidine derivatives	**27**	Unknown
**13**	Spirostanol steroidal saponins	**28**	Unknown
**14**	Fatty acids	**29**	Saponins
**15**	Steroidal alkaloids	**30**	Steroidal alkaloids

Table S3. Compounds annotated from cluster 20 by LC-DAD-MS data.

	RT (min)	Compound	UV (nm)	MF	MS (*m/z*) [M+H]$^+$	MS/MS (*m/z*)	MS (*m/z*) [M-H]$^-$	MS/MS (*m/z*)	Sample
**54**	16.2	*O*-galloyl-hexosyl myricetin	270, 350	$C_{28}H_{24}O_{17}$	633.1090	319, 153	631.0947	479, 316, 271, 169	*Psidium guineense*
**55**	18.4	*O*-galloyl-hexosyl quercetin	253, 270, 358	$C_{28}H_{24}O_{16}$	617.1141	303, 153	615.0971	463, 300, 169	*Psidium guineense*
**56**	22.7	di-*O*-hexosyl quercetin	265, 350	$C_{27}H_{30}O_{17}$	627.1546	465, 303, 267, 163	625.1410	300, 271, 255	*Byttneria filipes*
**57**	25.3	*O*-sinapoyl-hexosyl myricetin	270, 330, 355	$C_{32}H_{30}O_{17}$	687.1564	319, 207, 175	685.1413	479, 316, 271, 205	*Psidium guineense*
**58**	25.4	*O*-feruloyl-hexosyl myricetin	270, 330, 355	$C_{31}H_{28}O_{16}$	657.1448	319, 175	655.1327	316, 271	*Psidium guineense*
**59**	25.8	*O*-feruloyl di-hexosyl kaempferol	270, 330, 355	$C_{37}H_{38}O_{19}$	787.2078	287, 177	785.1948	-	*Sesbania virgata*
**60**	27.2	*O*-sinapoyl-hexosyl quercetin	265, 330, 357	$C_{32}H_{30}O_{16}$	671.1621	509, 491, 303, 207, 175	669.1443	300	*Sesbania virgata*
**61**	27.3	*O*-feruloyl-hexosyl quercetin	270, 330, 355	$C_{31}H_{28}O_{15}$	641.1481	-	639.1348	463, 300, 271, 255	*Sesbania virgata*
**62**	27.5	*O*-feruloyl-hexosyl quercetin	270, 330, 355	$C_{31}H_{28}O_{15}$	641.1499	-	639.1341	63, 300, 271, 255	*Sesbania virgata*
**63**	28.7	*O*-coumaroyl-hexosyl kaempferol	270, 315, 355	$C_{30}H_{26}O_{13}$	595.1446	287	593.1316	285	*Byttneria filipes*
**64**	29.0	*O*-feruloyl-hexosyl kaempferol	278, 320, 360	$C_{31}H_{28}O_{14}$	625.1538	287, 177	623.1402	284, 255, 227, 193, 161	*Byttneria filipes*

RT: retention time; MF: molecular formula. The MF was considered errors up 8 ppm and mSigma 30. *> 0.8 cos score.

Table S4. Compounds annotated from cluster 28 by LC-DAD-MS data.

	RT (min)	Compound	UV (nm)	MF	MS (*m/z*) [M+H]$^+$	MS/MS (*m/z*)	Sample
**71**	11.3	Unknown	292	$C_{15}H_{21}N3O_6$	340.1508	323, 305, 279, 241, 207, 189	*O. diospyrifolia*
**72**	13.2	Unknown	280, 307(sh)	$C_{16}H_{22}N_2O_7$	355.1507	249, 221, 207, 190	*O. diospyrifolia*
**73**	14.0	Unknown	279, 312 (sh)	$C_{16}H_{20}N_3O_7$	366.1301	297, 218, 207, 175, 163	*O. diospyrifolia*
**74**	15.2	Unknown	278	$C_{17}H_{24}N_2O_7$	369.1663	297, 235, 207, 157	*O. diospyrifolia*
**75**	17.4	Unknown	279	$C_{15}H_{21}N_3O_5$	324.1558	307, 271, 191, 175, 159	*O. diospyrifolia*

RT: retention time; MF: molecular formula. The MF was considered errors up 8 ppm and mSigma 30. *> 0.8 cos score.

Table S5. Compounds annotated by MZmine from LC-MS/MS data.

id	Compound_name	Adduct	score	MS (*m/z*)	MS/MS (*m/z*)	RT (min)	MF	Sample
972	Ellagic Acid	$[M+H]^+$	0.76	303.0136	275, 257, 247, 229, 201	18.5	$C_{14}H_6O_8$	28, 3, 45
2342	Isorhamnetin	$[M+H]^+$	0.78	317.0656	302, 285, 273, 229, 165, 153	30.46	$C_{16}H_{12}O_7$	26, 30, 43, 45, 6
2037	3-methylquercetin	$[M+H]^+$	0.89	317.0656	302, 274, 245, 229, 153	28.2	$C_{16}H_{12}O_7$	1, 2, 30, 6
792	Boldine	$[M+H]^+$	0.88	328.1543	297, 282, 265, 250, 237	16.4	$C_{19}H_{21}NO_4$	45, 46
704	Reticuline	$[M+H]^+$	0.73	330.1719	299, 267, 192, 177	15.3	$C_{19}H_{23}NO_4$	45, 46
2513	4',5,7-trihydroxy-3,6-dimethoxyflavone	$[M+H]^+$	0.72	331.0812	316, 315, 273	31.26	$C_{17}H_{14}O_7$	2, 30
1505	Pelargonidin *O*-hexoside	$[M]^+$	0.83	433.1124	271	22.32	$C_{21}H_{21}O_{10}$	16, 1, 21, 26, 2, 30, 31, 3, 45, 46, 4, 6, 7
1011	Apigenin-8-*C*-hexoside (vitexin)	$[M+H]^+$	0.78	433.1129	379, 361, 349, 337, 323, 313, 309, 295, 283, 165	25.55	$C_{21}H_{20}O_{10}$	16, 1, 26, 24, 28, 2, 30, 31, 33, 4, 4, 6, 7
1011	Apigenin-6-C-glucoside (isovitexin)	$[M+H]^+$	0.78	433.1129	379, 361, 349, 337, 323, 313, 309, 295, 283, 165	23.59	$C_{21}H_{20}O_{10}$	30, 31, 4, 7
1319	Quercetin O-pentoside	$[M+H]^+$	0.76	435.0920	303	20.5	$C_{20}H_{18}O_{11}$	16, 1, 26, 28, 31, 33, 46, 4, 6
1230	Quercetin O-pentoside	$[M+H]^+$	0.94	435.0927	303	20.8	$C_{20}H_{18}O_{11}$	16, 1, 26, 28, 31, 33, 46, 4, 6
1270	Quercetin O-pentoside	$[M+H]^+$	0.92	435.0927	303	21.07	$C_{20}H_{18}O_{11}$	16, 1, 26, 28, 31, 33, 46, 4, 6
1495	Apigenin *O*-glucuronide	$[M+H]^+$	0.81	447.0922	271	22.28	$C_{21}H_{18}O_11$	16, 22, 2, 30, 31, 3, 6, 7
827	Luteolin 6-*C*-hexoside	$[M+H]^+$	0.93	449.1078	413, 395, 353, 329, 299	17.31	$C_{21}H_{20}O_{11}$	16, 1, 26, 24, 28, 2, 30, 31, 33, 31, 45, 4
1301	Luteolin *O*-hexoside	$[M+H]^+$	0.99	449.1078	287	23.53	$C_{21}H_{20}O_{11}$	16, 1, 26, 2, 31, 31, 33, 3, 45, 46, 4, 6, 7
1455	Quercetin *O*-deoxyhexoside	$[M+H]^+$	0.96	449.1084	303	21.84	$C_{21}H_{20}O_{11}$	16, 1, 21, 22, 26, 28, 2, 30, 31, 33, 45, 46, 6, 7
1301	Kaempferol 3-*O*-hexoside	$[M+H]^+$	0.99	449.1100	287	21	$C_{21}H_{20}O_{11}$	16, 1, 26, 24, 2, 30, 21, 45, 46, 4, 6
876	Myricetin *O*-pentoside	$[M+H]^+$	0.84	451.0871	319	18.5	$C_{20}H_{18}O_{12}$	26, 28, 31, 33
604	Isookanin *O*-hexoside	$[M+H]^+$	0.93	451.1240	289, 271, 163, 153	14.8	$C_{21}H_{22}O_{11}$	46, 4

604	Okanin *O*-hexoside	$[M+H]^+$	0.91	451.1240	289, 271, 243, 163, 153	21.45	$C_{21}H_{22}O_{11}$	22, 31, 4
2137	Oroxylin *O*-glucuronide	$[M+H]^+$	0.73	461.1080	285, 270, 242	28.9	$C_{22}H_{20}O_{11}$	2, 3
1127	Kaempferol *O*-glucuronide	$[M+H]^+$	0.91	463.0871	287	19.75	$C_{21}H_{18}O_{12}$	16, 2, 30, 3, 4, 7
1608	Peonidin *O*-hexoside	$[M]^+$	0.92	463.1229	301	23.36	$C_{22}H_{23}O_{11}$	16, 1, 22, 2, 30, 31, 33, 3, 45, 4, 6, 7
1016	Myricitin *O*-deoxyhexoside	$[M+H]^+$	0.98	465.1034	319	18.89	$C_{21}H_{20}O_{12}$	16, 1, 22, 26, 24, 28, 2, 30, 31, 4, 46, 7
1065	Quercetin *O*-hexoside	$[M+H]^+$	0.99	465.1034	303	19.67	$C_{21}H_{20}O_{12}$	16, 1, 21, 22, 26, 28, 2, 30, 31, 33, 43, 46
3951	18β-glycyrrhetinic acid	$[M+H]^+$	0.72	471.3453	389, 285, 201	38.35	$C_{30}H_{46}O_4$	26, 24, 28, 30
1601	Diosmetin *O*-glucuronide	$[M+H]^+$	0.95	477.1024	301, 286	23.31	$C_{22}H_{20}O_{12}$	2, 30, 3, 7
2218	Cirsimaritin *O*-hexoside	$[M+H]^+$	0.77	477.1391	315, 300, 269	29.56	$C_{23}H_{24}O_{11}$	1, 28
1008	quercetin *O*-glucuronide	$[M+H]^+$	0.84	479.0820	303, 229, 163, 153	18.72	$C_{21}H_{18}O_{13}$	2
1768	Petunidin *O*-hexoside	$[M]^+$	0.87	479.1179	317, 302	22.11	$C_{22}H_{23}O_{12}^+$	16, 1, 22, 26, 2, 33, 43, 45, 46, 6
777	Myricetin *O*-hexoside	$[M+H]^+$	0.98	481.0982	319, 273, 263, 245, 217, 165, 153	16.87	$C_4H_9NO_2$	16, 1, 28, 31, 33, 45, 7
2200	Afrormosin *O*-hexoside-malonate	$[M+H]^+$	0.79	547.1443	299, 284, 175, 151	29.42	$C_{26}H_{26}O_{13}$	1, 4
1748	*O*-caffeoyl *O*-sinapoyl quinic acid	$[M+H]^+$	0.93	561.1603	207, 175, 163	25.81	$C_{27}H_{28}O_{13}$	30, 43
1843	*O*-caffeoyl *O*-sinapoyl quinic acid	$[M+H]^+$	0.93	561.1603	207, 179, 175, 163	24.96	$C_{27}H_{28}O_{13}$	16, 30, 43
1020	Isovitexin *O*-pentoside	$[M+H]^+$	0.77	565.1552	529, 475, 437, 355, 349, 325, 307, 245	18.9	$C_{266}H_{28}O_{14}$	16, 45, 4
1099	Isovitexin *O*-deoxyhexoside	$[M+H]^+$	0.91	579.1708	433, 337, 313, 283, 246	22.37	$C_{27}H_{30}O_{14}$	24, 31, 45, 4, 7
1044	Vitexin *O*-deoxyhexoside	$[M+H]^+$	0.87	579.1714	451, 397, 379, 337, 323, 313, 283	19.01	$C_{27}H_{30}O_{14}$	16, 1, 26, 24, 30, 31, 4, 7
2340	Tricoumaroyl spermidine	$[M+H]^+$	0.81	584.2795	438, 204	30.41	$C_{34}H_{37}N_3O_6$	28
2104	Luteolin *O*-coumaroyl *O*-hexoside	$[M+H]^+$	0.96	595.1446	287.0000	28.68	$C_{30}H_{26}O_{13}$	16, 01, 24, 2, 30, 3, 46, 4
1832	Apigenin 8-*C*-hexosy *O*-hexoside	$[M+H]^+$	0.87	595.1629	475, 415, 397, 379, 313, 163	25.68	$C_{27}H_{30}O_{15}$	16, 1, 30
1386	Cyanidin *O*-deoxyhexosyl *O*-hexoside	$[M]^+$	1.00	595.1652	287	21.42	$C_{27}H_{31}O_{15}$	16, 1, 22, 26, 24, 2, 30, 33, 43, 6, 7
683	Apigenin di-*C*-6,8-hexoside	$[M+H]^+$	0.86	595.1657	541, 505, 457, 409, 379, 337, 307	15.59	$C_{27}H_{30}O_{15}$	16, 1, 26, 28, 2, 30, 31, 3, 45, 4, 6

774	Vitexin *O*-hexoside	$[M+H]^+$	0.88	595.1657	361, 313, 283	16.85	$C_{27}H_{30}O_{15}$	1, 22, 28, 30, 31, 3, 45, 4, 6
1132	Quercetin di-*O*-deoxyhexoside	$[M+H]^+$	0.77	595.1657	303, 165, 153	19.81	$C_{27}H_{30}O_{15}$	16, 1, 22, 26, 28, 30, 33, 3, 45, 46, 4, 6, 7
2078	Datiscetin *O*-deoxyhexosyl O-hexoside	$[M+H]^+$	0.82	595.1657	287, 165, 147	28.52	$C_{27}H_{30}O_{15}$	16, 1, 24, 2, 30, 46, 6
2175	Kaempferol *O*-hexosyl-*O*-deoxyhexoside	$[M+H]^+$	0.90	595.1657	287, 239, 163, 147	29.15	$C_{27}H_{30}O_{15}$	16, 1, 2, 30, 4
1072	Quercetin *O*-kexosyl-deoxyhexoside	$[M+H]^+$	0.96	611.1607	465, 303, 255, 153	19.37	$C_{27}H_{30}O_{16}$	16, 1, 21, 22, 26, 24, 28, 2, 33, 3, 43, 45, 46, 4, 6
1072	Rutin*	$[M+H]^+$	0.96	611.1614	465, 303, 153	18.24	$C_{27}H_{30}O_{16}$	16, 1, 26, 24, 2, 30, 46
1415	Isorhamnetin *O*-deoxyhexosyl O-hexoside	$[M+H]^+$	0.84	625.1763	317, 261	22.09	$C_{28}H_{32}O_{16}$	1, 26, 24, 46, 4, 6
1246	Kaempferol *O*-hexosyl *O*-pentosyl-deoxyhexoside	$[M+H]^+$	0.94	727.2080	303	20.61	$C_{32}H_{38}O_{19}$	26, 33, 45, 46
1040	Kaempferol *O*-hexosyl di-*O*-deoxyhexoside	$[M+H]^+$	0.96	741.2237	287	19.0	$C_{33}H_{40}O_{19}$	1, 26, 24, 30, 46
825	Cyanidin di-*O*-hexosyl-*O*-pentoside	$[M]^+$	0.74	743.2024	449, 303, 261, 229, 171	17.37	$C_{32}H_{39}O_{20}$	26, 33, 45, 6
874	Quercetin di-*O*-deoxyhexosyl-*O*-hexoside	$[M+H]^+$	0.89	757.2186	303, 255, 239, 171, 153	17.67	$C_{33}H_{40}O_{20}$	1, 26, 24, 30, 45, 46, 4
751	Quercetin di-*O*-hexosyl *O*-pentoside	$[M+H]^+$	0.87	759.1978	465, 303, 153	16.53	$C_{32}H_{38}O_{21}$	45
2938	Triterpenoid saponin /O-glucuronyl *O*-hexosyl oleanolic acid (calenduloside F)	$[M+Na]^+$	0.91	817.4344	641, 623, 439	32.9	$C_{42}H_{66}O_{14}$	1, 21, 24, 30
3205	Triterpenoid saponin (Soyasaponin I)	$[M+H]^+$	0.94	943.5261	599, 581, 441, 423, 365, 261, 217	33.97	$C_{48}H_{78}O_{18}$	1, 24, 33, 45, 4, 6, 7
2851	Triterpenoid saponin (Putranoside C)	$[M+Na]^+$	0.71	963.4924	817, 641, 345, 187	32.69	$C_{48}H_{76}O_{18}$	1, 21, 46
2676	Triterpenoid saponin (calenduloside H)	$[M+Na]^+$	0.87	979.4872	817, 439, 249, 191	32.06	$C_{48}H_{76}O_{19}$	1, 21

RT: retention time; MF: molecular formula. *confirmed by injection of authentic standard. Samples: 1: *Sesbania virgata*; 2: *Centratherum punctatum*; 3: *Lantana canescens*; 4: *Melanthera latifólia*; 6: *Aeschynomene denticulata*; 7: *Echinodorus paniculatus*; 16: *Byttneria filipes*; 21: *Aspilia latissima*; 22: *Erythroxylum anguifugum*; 24: *Tocoyena formosa*; 26: *Diospyros tetrasperma*; 28: *Psidium guineense*; 30: *Vitex cymosa*; 31: *Astronium fraxinifolium*; 33: *Coccoloba ochreolata*; 43: *Solanum glaucophyllum*; 45: *Paullinia pinnata*; 46: *Ocotea diospyrifolia*

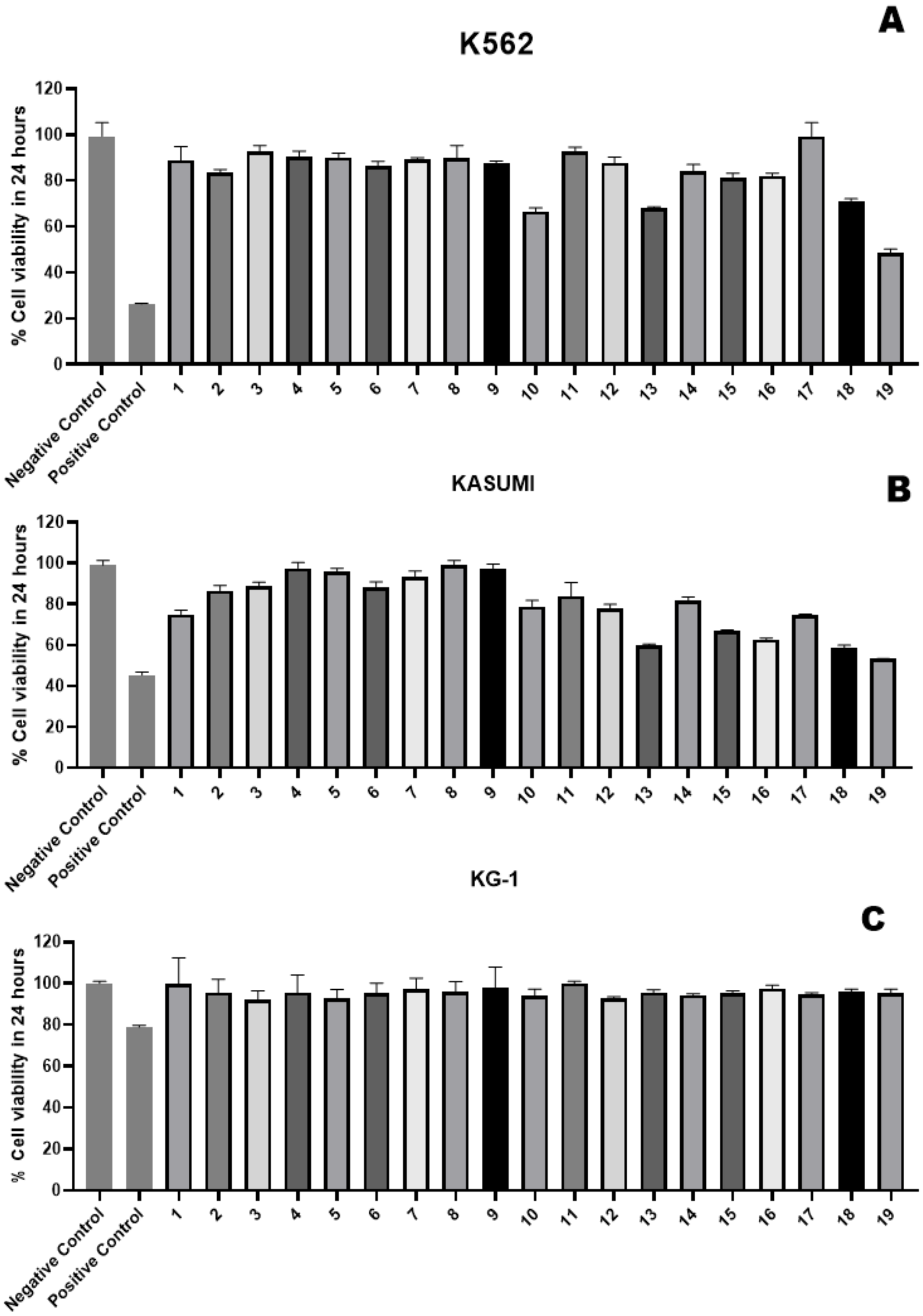

Figure S1. Cell proliferation and cytotoxic screening of the polar extracts on K562 (A), Kasumi-1 (B), and KG-1 (C) leukemia cell lines at 10µg/mL for 24h. Extracts from the following species*:* *Sesbania virgata* (1), *Cenetrratherum punctatum* (2), *Lantana canescens* (3), *Melanthera latifolia* (4), *Aeschynomene denticulata* (5), *Echinodorus paniculatus* (6), *Byttemeria filipes* (7), *Aspilia latissima* – aerial parts (AP) (8), *A. latissima* - roots (9), *Erythroxylum anguifugum* (10), *Tocoyena formosa* (11), *Diospyros tetrasperma* (12), *Psidium guineense* (13), *Vitex cymosa* (14), *Astronium fraxinifolium* (15), *Coccoloba ochreolata* (16), *Solanum glaucophyllum* (17), *Paullinia pinnata* (18), and *Ocotea diospyrifolia* (19). were evaluated. Doxorubicin was used as positive control. Results were expressed as mean ± standard deviation (SD).

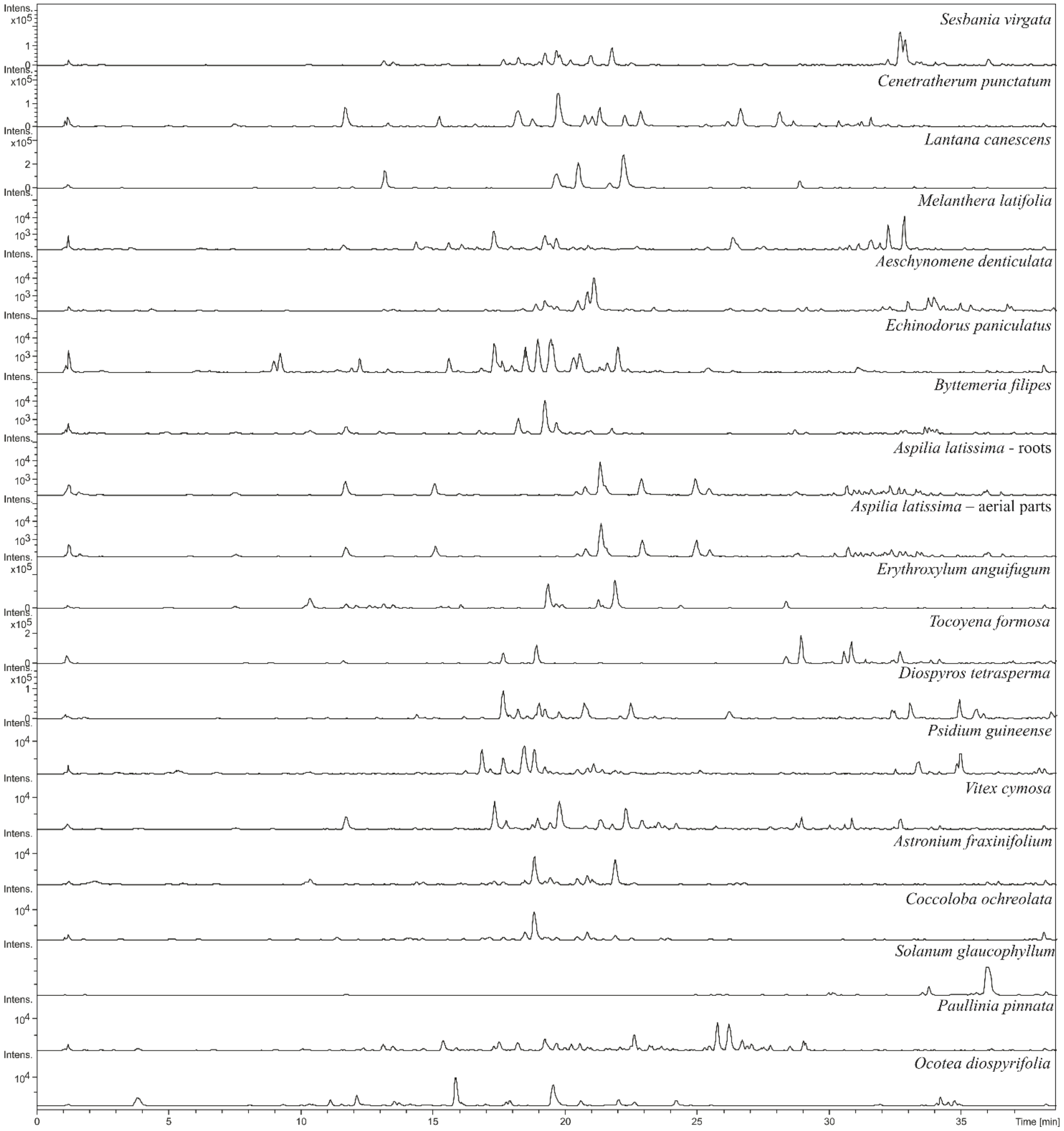

Figure S2. Base peak chromatogram obtained in positive ion mode from *Sesbania virgata*, *Cenetrratherum punctatu*, *Lantana canescens*, *Melanthera latifolia*, *Aeschynomene denticulata*, *Echinodorus paniculatus*, *Byttemeria filipes*, *Aspilia latissima* – aerial parts, *A. latissima* - roots, *Erythroxylum anguifugum*, *Tocoyena formosa*, *Diospyros tetrasperma*, *Psidium guineense*, *Vitex cymosa*, *Astronium fraxinifolium*, *Coccoloba ochreolata*, *Solanum glaucophyllum*, *Paullinia pinnata*, and *Ocotea diospyrifolia*.

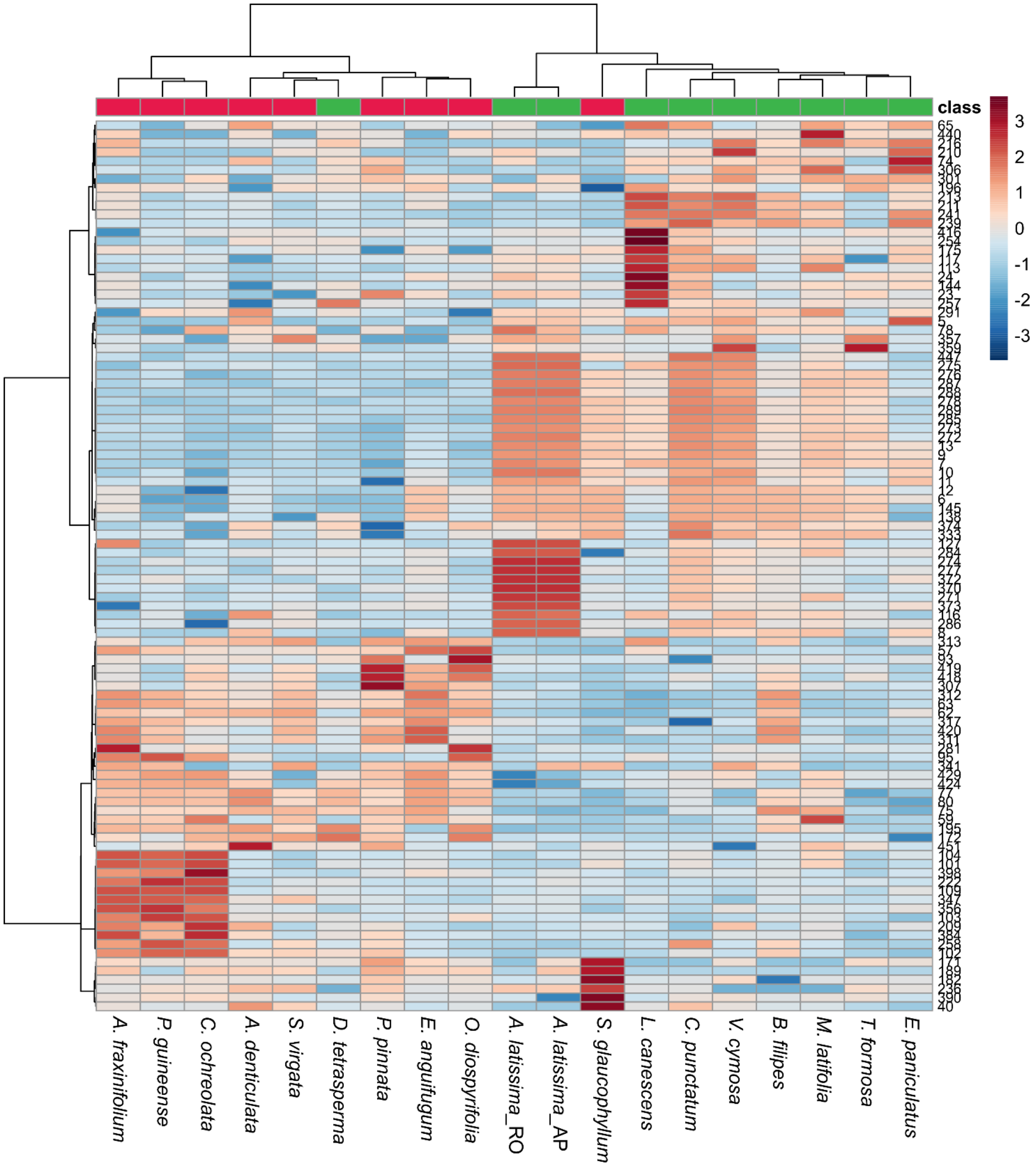

Figure S3. Heatmap and hierarchical clustering (HCA) of ion intensities of the top 100 features. The samples were classified in the groups active (red) and inactive (green) against leukemia cancer cells. AP: aerial parts; RO: roots.

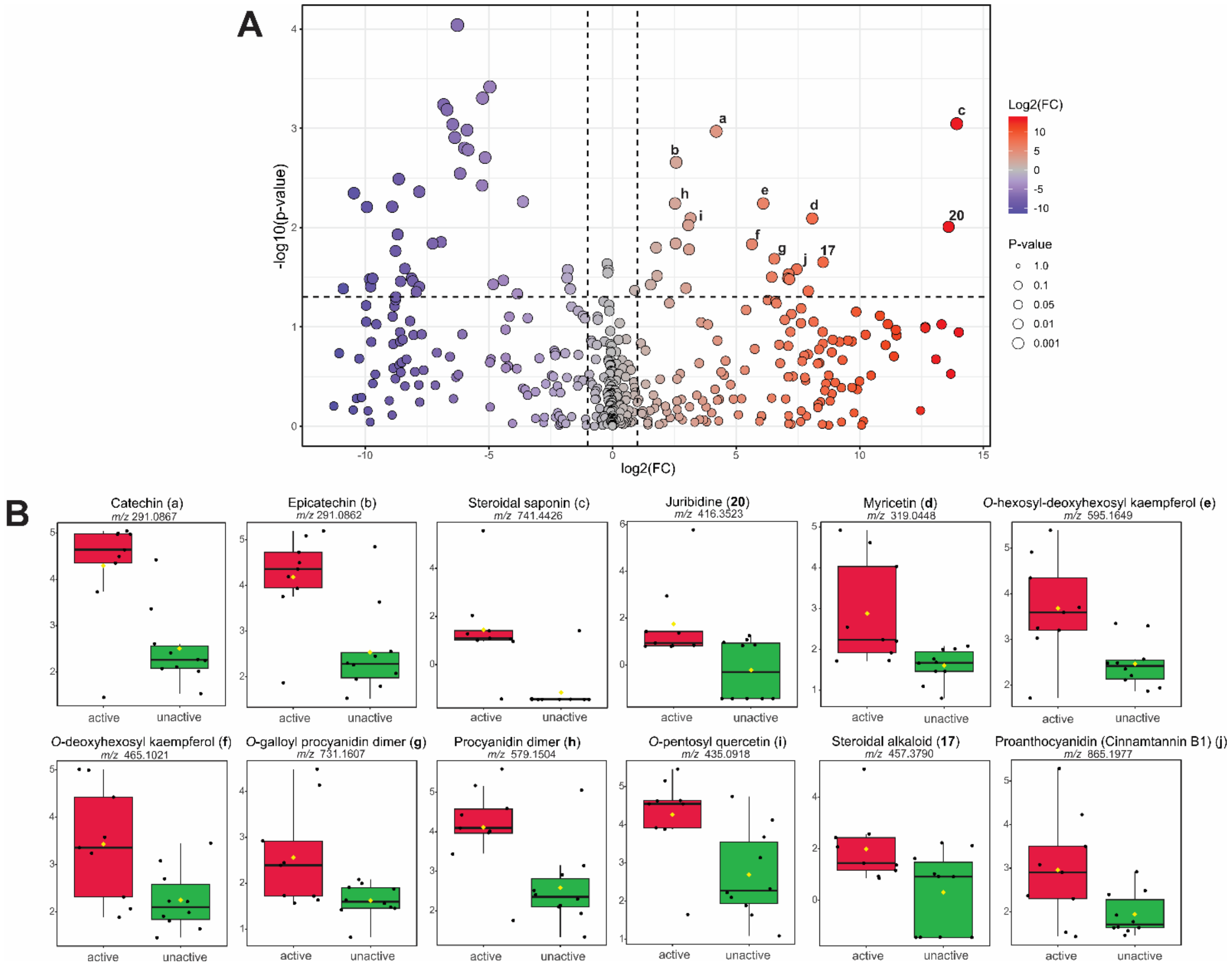

Figure S4. Volcano plot displaying the differences in features between the active (right quadrant) and inactive extracts (left quadrant). The x and y axes represent the fold change (FC) and P values of the features highlighted showed p≤0.05 and they were illustrated in the box plots below (**A**). Box plots from the annotated features that revealed p≤0.05 for the active samples (**B**).

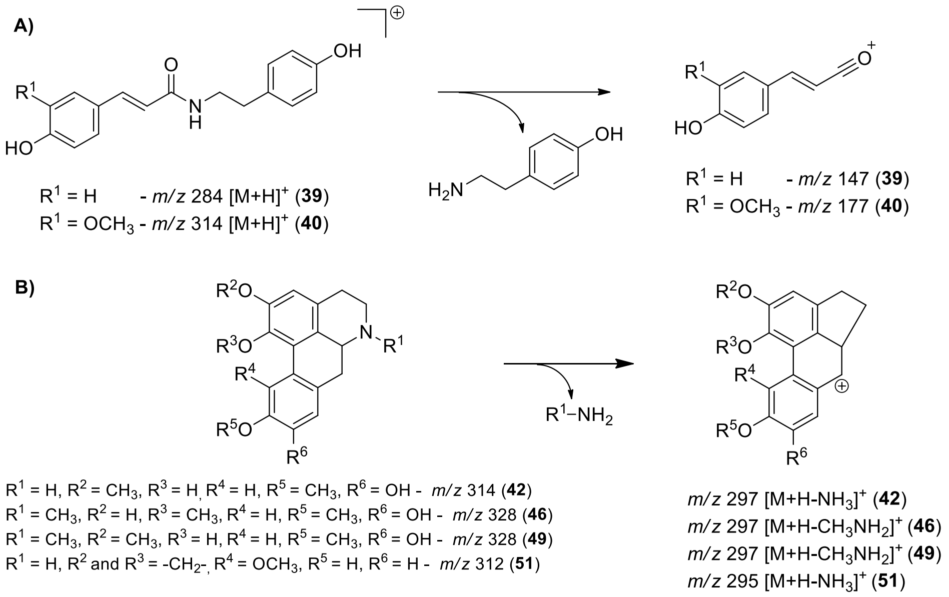

Figure S5. Fragmentation pathway for tyramine derivatives (**A**) and aporphine alkaloids (**B**).

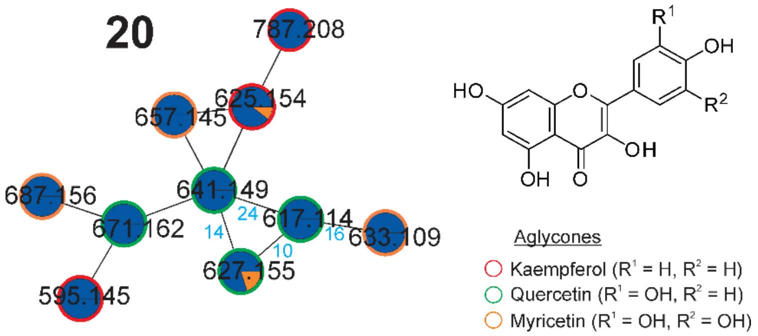

Figure S6. Cluster of *O*-glycosyl-phenylpropanoyl/gallolyl flavonols (cluster 20). The annotation of features is described in **Table S3**.

Parameters applied for data Processing by MZmine


```
<?xml version="1.0" encoding="UTF-8"?><batch mzmine_version="4.5.0">
  <batchstep method="io.github.mzmine.modules.io.import_rawdata_all.AllSpectralDataImportModule"
parameter_version="1">
      <parameter name="File names">
      </parameter>
      <parameter name="Advanced import" selected="false">
        <parameter name="Scan filters" selected="true">
          <parameter name="Scan number"/>
          <parameter name="Base Filtering Integer"/>
          <parameter name="Retention time"/>
          <parameter name="Mobility"/>
          <parameter name="MS level filter" selected="All MS levels">1</parameter>
          <parameter name="Scan definition"/>
          <parameter name="Polarity">Any</parameter>
          <parameter name="Spectrum type">ANY</parameter>
        </parameter>
        <parameter name="Crop MS1 m/z" selected="false"/>
        <parameter name="MS1 detector (Advanced)" selected="false" selected_item="Factor of lowest signal">
          <module name="Factor of lowest signal">
            <parameter name="Noise factor">2.5</parameter>
          </module>
          <module name="Auto">
            <parameter name="Noise level">1000.0</parameter>
          </module>
          <module name="Centroid">
            <parameter name="Noise level"/>
          </module>
          <module name="Exact mass">
            <parameter name="Noise level"/>
          </module>
          <module name="Local maxima">
            <parameter name="Noise level"/>
          </module>
          <module name="Recursive threshold">
            <parameter name="Noise level"/>
            <parameter name="Min m/z peak width"/>
            <parameter name="Max m/z peak width"/>
          </module>
          <module name="Wavelet transform">
            <parameter name="Noise level"/>
            <parameter name="Scale level"/>
            <parameter name="Wavelet window size (%)"/>
          </module>
        </parameter>
        <parameter name="MS2 detector (Advanced)" selected="false" selected_item="Factor of lowest signal">
          <module name="Factor of lowest signal">
            <parameter name="Noise factor">2.5</parameter>
          </module>
          <module name="Auto">
            <parameter name="Noise level">1000.0</parameter>
          </module>
          <module name="Centroid">
            <parameter name="Noise level"/>
          </module>
          <module name="Exact mass">
            <parameter name="Noise level"/>
          </module>
          <module name="Local maxima">
            <parameter name="Noise level"/>
          </module>
```

```
<module name="Recursive threshold">
<parameter name="Noise level"/>
<parameter name="Min m/z peak width"/>
<parameter name="Max m/z peak width"/>
</module>
<module name="Wavelet transform">
<parameter name="Noise level"/>
<parameter name="Scale level"/>
<parameter name="Wavelet window size (%)"/>
</module>
</parameter>
<parameter name="Denormalize fragment scans (traps)">false</parameter>
</parameter>
<parameter name="Metadata file" selected="false"/>
<parameter name="Sort and color">true</parameter>
<parameter name="Spectral library files"/>
</batchstep>
<batchstep method="io.github.mzmine.modules.dataprocessing.featdet_massdetection.MassDetectionModule" parameter_version="1">
<parameter name="Raw data files" type="BATCH_LAST_FILES"/>
<parameter name="Scan filters" selected="true">
<parameter name="Scan number"/>
<parameter name="Base Filtering Integer"/>
<parameter name="Retention time">
<min>0.01</min>
<max>46.2</max>
</parameter>
<parameter name="Mobility"/>
<parameter name="MS level filter" selected="MS1, level = 1">1</parameter>
<parameter name="Scan definition"/>
<parameter name="Polarity">Any</parameter>
<parameter name="Spectrum type">ANY</parameter>
</parameter>
<parameter name="Scan types (IMS)">All scan types</parameter>
<parameter name="Denormalize fragment scans (traps)">false</parameter>
<parameter name="Mass detector" selected_item="Centroid">
<module name="Factor of lowest signal">
<parameter name="Noise factor">2.5</parameter>
</module>
<module name="Auto">
<parameter name="Noise level">1000.0</parameter>
</module>
<module name="Centroid">
<parameter name="Noise level">200.0</parameter>
</module>
<module name="Exact mass">
<parameter name="Noise level"/>
</module>
<module name="Local maxima">
<parameter name="Noise level"/>
</module>
<module name="Recursive threshold">
<parameter name="Noise level"/>
<parameter name="Min m/z peak width"/>
<parameter name="Max m/z peak width"/>
</module>
<module name="Wavelet transform">
<parameter name="Noise level"/>
<parameter name="Scale level"/>
<parameter name="Wavelet window size (%)"/>
</module>
</parameter>
```

```
    </batchstep>
    <batchstep method="io.github.mzmine.modules.dataprocessing.featdet_massdetection.MassDetectionModule" parameter_version="1">
        <parameter name="Raw data files" type="BATCH_LAST_FILES"/>
        <parameter name="Scan filters" selected="true">
            <parameter name="Scan number"/>
            <parameter name="Base Filtering Integer"/>
            <parameter name="Retention time">
                <min>0.01</min>
                <max>46.2</max>
            </parameter>
            <parameter name="Mobility"/>
            <parameter name="MS level filter" selected="MS2, level = 2">1</parameter>
            <parameter name="Scan definition"/>
            <parameter name="Polarity">Any</parameter>
            <parameter name="Spectrum type">ANY</parameter>
        </parameter>
        <parameter name="Scan types (IMS)">All scan types</parameter>
        <parameter name="Denormalize fragment scans (traps)">false</parameter>
        <parameter name="Mass detector" selected_item="Centroid">
            <module name="Factor of lowest signal">
                <parameter name="Noise factor">2.5</parameter>
            </module>
            <module name="Auto">
                <parameter name="Noise level">1000.0</parameter>
            </module>
            <module name="Centroid">
                <parameter name="Noise level">100.0</parameter>
            </module>
            <module name="Exact mass">
                <parameter name="Noise level"/>
            </module>
            <module name="Local maxima">
                <parameter name="Noise level"/>
            </module>
            <module name="Recursive threshold">
                <parameter name="Noise level"/>
                <parameter name="Min m/z peak width"/>
                <parameter name="Max m/z peak width"/>
            </module>
            <module name="Wavelet transform">
                <parameter name="Noise level"/>
                <parameter name="Scale level"/>
                <parameter name="Wavelet window size (%)"/>
            </module>
        </parameter>
    </batchstep>
    <batchstep
method="io.github.mzmine.modules.dataprocessing.featdet_adapchromatogrambuilder.ModularADAPChromatogram
BuilderModule" parameter_version="1">
        <parameter name="Raw data files" type="BATCH_LAST_FILES"/>
        <parameter name="Scan filters" selected="true">
            <parameter name="Scan number"/>
            <parameter name="Base Filtering Integer"/>
            <parameter name="Retention time">
                <min>0.5</min>
                <max>46.0</max>
            </parameter>
            <parameter name="Mobility"/>
            <parameter name="MS level filter" selected="MS1, level = 1">1</parameter>
            <parameter name="Scan definition"/>
            <parameter name="Polarity">Any</parameter>
```

```xml
        <parameter name="Spectrum type">ANY</parameter>
      </parameter>
      <parameter name="Minimum consecutive scans">4</parameter>
      <parameter name="Minimum intensity for consecutive scans">400.0</parameter>
      <parameter name="Minimum absolute height">200.0</parameter>
      <parameter name="m/z tolerance (scan-to-scan)">
        <absolutetolerance>0.002</absolutetolerance>
        <ppmtolerance>10.0</ppmtolerance>
      </parameter>
      <parameter name="Suffix">chromatograms</parameter>
      <parameter name="Allow single scan chromatograms"/>
    </batchstep>
    <batchstep
method="io.github.mzmine.modules.dataprocessing.featdet_chromatogramdeconvolution.minimumsearch.MinimumSearchFeatureResolverModule" parameter_version="2">
      <parameter name="Feature lists" type="BATCH_LAST_FEATURELISTS"/>
      <parameter name="Suffix">resolved</parameter>
      <parameter name="Original feature list">KEEP</parameter>
      <parameter name="MS/MS scan pairing" selected="true">
        <parameter name="MS1 to MS2 precursor tolerance (m/z)">
          <absolutetolerance>0.02</absolutetolerance>
          <ppmtolerance>10.0</ppmtolerance>
        </parameter>
        <parameter name="Retention time filter" selected="Use feature edges" unit="MINUTES">0.2</parameter>
        <parameter name="Minimum relative feature height" selected="true">0.25</parameter>
        <parameter name="Minimum required signals" selected="true">1</parameter>
        <parameter name="Limit by ion mobility edges">false</parameter>
        <parameter name="Merge MS/MS spectra (TIMS)">false</parameter>
        <parameter name="Minimum detections in IMS dimension">2</parameter>
        <parameter name="Advanced" selected="false">
          <parameter name="Minimum signal intensity (absolute, TIMS)" selected="false">250.0</parameter>
          <parameter name="Minimum signal intensity (relative, TIMS)" selected="true">0.01</parameter>
        </parameter>
      </parameter>
      <parameter name="Dimension">Retention time</parameter>
      <parameter name="Chromatographic threshold">0.85</parameter>
      <parameter name="Minimum search range RT/Mobility (absolute)">0.05</parameter>
      <parameter name="Minimum relative height">0.0</parameter>
      <parameter name="Minimum absolute height">1000.0</parameter>
      <parameter name="Min ratio of peak top/edge">1.7</parameter>
      <parameter name="Peak duration range (min/mobility)">
        <min>0.0</min>
        <max>10.0</max>
      </parameter>
      <parameter name="Minimum scans (data points)">3</parameter>
    </batchstep>
    <batchstep method="io.github.mzmine.modules.dataprocessing.filter_isotopegrouper.IsotopeGrouperModule" parameter_version="1">
      <parameter name="Feature lists" type="BATCH_LAST_FEATURELISTS"/>
      <parameter name="Name suffix">deisotoped</parameter>
      <parameter name="m/z tolerance (intra-sample)">
        <absolutetolerance>0.001</absolutetolerance>
        <ppmtolerance>8.0</ppmtolerance>
      </parameter>
      <parameter name="Retention time tolerance" unit="MINUTES">0.04</parameter>
      <parameter name="Mobility tolerance" selected="false"/>
      <parameter name="Monotonic shape">true</parameter>
      <parameter name="Maximum charge">2</parameter>
      <parameter name="Representative isotope">Most intense</parameter>
      <parameter name="Never remove feature with MS2">true</parameter>
      <parameter name="Original feature list">KEEP</parameter>
    </batchstep>
```

```
    <batchstep method="io.github.mzmine.modules.dataprocessing.align_join.JoinAlignerModule"
parameter_version="1">
        <parameter name="Feature lists" type="BATCH_LAST_FEATURELISTS"/>
        <parameter name="Feature list name">Aligned feature list</parameter>
        <parameter name="m/z tolerance (sample-to-sample)">
            <absolutetolerance>0.002</absolutetolerance>
            <ppmtolerance>8.0</ppmtolerance>
        </parameter>
        <parameter name="Weight for m/z">3.0</parameter>
        <parameter name="Retention time tolerance" unit="MINUTES">0.25</parameter>
        <parameter name="Weight for RT">1.0</parameter>
        <parameter name="Mobility tolerance" selected="false"/>
        <parameter name="Mobility weight">1.0</parameter>
        <parameter name="Require same charge state">false</parameter>
        <parameter name="Require same ID">false</parameter>
        <parameter name="Compare isotope pattern" selected="false">
            <parameter name="Isotope m/z tolerance">
                <absolutetolerance>0.001</absolutetolerance>
                <ppmtolerance>5.0</ppmtolerance>
            </parameter>
            <parameter name="Minimum absolute intensity">0.0</parameter>
            <parameter name="Minimum score">0.0</parameter>
        </parameter>
        <parameter name="Compare spectra similarity" selected="false">
            <parameter name="Spectral m/z tolerance">
                <absolutetolerance>0.001</absolutetolerance>
                <ppmtolerance>10.0</ppmtolerance>
            </parameter>
            <parameter name="MS level">2</parameter>
            <parameter name="Compare spectra similarity" selected_item="Weighted cosine similarity">
                <module name="Weighted cosine similarity">
                    <parameter name="Weights">MassBank (mz^2 * I^0.5)</parameter>
                    <parameter name="Minimum  cos similarity">0.7</parameter>
                    <parameter name="Handle unmatched signals">KEEP ALL AND MATCH TO ZERO</parameter>
                </module>
                <module name="Composite cosine identity (e.g., GC-EI-MS; similar to NIST search)">
                    <parameter name="Weights">MassBank (mz^2 * I^0.5)</parameter>
                    <parameter name="Minimum  cos similarity">0.7</parameter>
                    <parameter name="Handle unmatched signals">KEEP ALL AND MATCH TO ZERO</parameter>
                </module>
            </parameter>
        </parameter>
        <parameter name="Original feature list">KEEP</parameter>
    </batchstep>
    <batchstep
method="io.github.mzmine.modules.dataprocessing.filter_blanksubtraction.FeatureListBlankSubtractionModule"
parameter_version="1">
        <parameter name="Aligned feature list" type="BATCH_LAST_FEATURELISTS"/>
        <parameter name="Blank/Control raw data files" type="BATCH_LAST_FILES"/>
        <parameter name="Minimum # of detection in blanks">1</parameter>
        <parameter name="Quantification">Height</parameter>
        <parameter name="Ratio type">MAXIMUM</parameter>
        <parameter name="Fold change increase" selected="false">3.0</parameter>
        <parameter name="Keep or remove features (of rows) below fold change">REMOVE - Only keep features above
fold change</parameter>
        <parameter name="Create secondary list of subtracted features">false</parameter>
        <parameter name="Suffix">subtracted</parameter>
    </batchstep>
    <batchstep
method="io.github.mzmine.modules.dataprocessing.gapfill_peakfinder.multithreaded.MultiThreadPeakFinderModule"
parameter_version="1">
        <parameter name="Feature lists" type="BATCH_LAST_FEATURELISTS"/>
```

```
        <parameter name="Name suffix">gap-filled</parameter>
        <parameter name="Intensity tolerance">0.2</parameter>
        <parameter name="m/z tolerance (sample-to-sample)">
            <absolutetolerance>0.002</absolutetolerance>
            <ppmtolerance>8.0</ppmtolerance>
        </parameter>
        <parameter name="Retention time tolerance" unit="MINUTES">0.2</parameter>
        <parameter name="Minimum scans (data points)">2</parameter>
        <parameter name="Original feature list">KEEP</parameter>
    </batchstep>
    <batchstep method="io.github.mzmine.modules.dataprocessing.norm_linear.LinearNormalizerModule"
parameter_version="1">
        <parameter name="Feature lists" type="BATCH_LAST_FEATURELISTS"/>
        <parameter name="Name suffix">normalized</parameter>
        <parameter name="Normalization type">Average intensity</parameter>
        <parameter name="Feature measurement type">Area</parameter>
        <parameter name="Original feature list">KEEP</parameter>
    </batchstep>
    <batchstep
method="io.github.mzmine.modules.dataprocessing.id_spectral_library_match.SpectralLibrarySearchModule"
parameter_version="3">
        <parameter name="Spectral libraries" type="ALL_IMPORTED"/>
        <parameter name="Feature lists" type="BATCH_LAST_FEATURELISTS"/>
        <parameter name="Merge &amp; select fragment scans" selected_item="simple_merged">
            <module name="simple_merged">
                <parameter name="Presets">representative_scans</parameter>
                <parameter name="Merging m/z tolerance">
                    <absolutetolerance>0.008</absolutetolerance>
                    <ppmtolerance>25.0</ppmtolerance>
                </parameter>
            </module>
            <module name="preset_merged">
                <parameter name="Presets">representative_scans</parameter>
                <parameter name="Merging m/z tolerance">
                    <absolutetolerance>0.008</absolutetolerance>
                    <ppmtolerance>25.0</ppmtolerance>
                </parameter>
                <parameter name="Merge">
                    <selected>Across samples</selected>
                </parameter>
                <parameter name="Intensity merge mode">MAXIMUM</parameter>
            </module>
            <module name="input_scans">
                <parameter name="Select input scans">most_intense_across_samples</parameter>
            </module>
        </parameter>
        <parameter name="MS level filter" selected="MS2, level = 2">3</parameter>
        <parameter name="Precursor m/z tolerance">
            <absolutetolerance>0.02</absolutetolerance>
            <ppmtolerance>8.0</ppmtolerance>
        </parameter>
        <parameter name="Spectral m/z tolerance">
            <absolutetolerance>0.0015</absolutetolerance>
            <ppmtolerance>10.0</ppmtolerance>
        </parameter>
        <parameter name="Remove precursor">true</parameter>
        <parameter name="Minimum  matched signals">4</parameter>
        <parameter name="Similarity" selected_item="Weighted cosine similarity">
            <module name="Weighted cosine similarity">
                <parameter name="Weights">MassBank (mz^2 * I^0.5)</parameter>
                <parameter name="Minimum  cos similarity">0.7</parameter>
                <parameter name="Handle unmatched signals">KEEP ALL AND MATCH TO ZERO</parameter>
```

```xml
</module>
<module name="Composite cosine identity (e.g., GC-EI-MS; similar to NIST search)">
<parameter name="Weights">MassBank (mz^2 * I^0.5)</parameter>
<parameter name="Minimum  cos similarity">0.7</parameter>
<parameter name="Handle unmatched signals">KEEP ALL AND MATCH TO ZERO</parameter>
</module>
</parameter>
<parameter name="Advanced" selected="false">
<parameter name="Retention time tolerance" selected="false"/>
<parameter name="CCS tolerance [%]" selected="false">0.05</parameter>
<parameter name="13C deisotoping" selected="false">
<parameter name="m/z tolerance"/>
<parameter name="Monotonic shape">false</parameter>
<parameter name="Maximum charge"/>
</parameter>
<parameter name="Min matched isotope signals" selected="false">3</parameter>
<parameter name="Crop spectra to m/z overlap">false</parameter>
</parameter>
</batchstep>
<batchstep method="io.github.mzmine.modules.io.export_network_graphml.NetworkGraphMlExportModule" parameter_version="1">
<parameter name="Feature lists" type="BATCH_LAST_FEATURELISTS"/>
<parameter name="Filename">
<current_file relative_path="..\..\artigo\ACS omega\FBN_GNPS.graphml">C:\Users\denis\OneDrive\Documents\Documentos_Denise\Suni\artigo\ACS omega\FBN_GNPS.graphml</current_file>
<last_file>C:\Users\denis\OneDrive\Documents\Documentos_Denise\Suni\artigo\ACS omega\FBN_GNPS.graphml</last_file>
</parameter>
</batchstep>
</batch>
```
